# Generational distribution of a *Candida glabrata* population: Resilient old cells prevail, while younger cells dominate in the vulnerable host

**DOI:** 10.1371/journal.ppat.1006355

**Published:** 2017-05-10

**Authors:** Tejas Bouklas, Luz Alonso-Crisóstomo, Tamás Székely, Elizabeth Diago-Navarro, Erika P. Orner, Kalie Smith, Mansa A. Munshi, Maurizio Del Poeta, Gábor Balázsi, Bettina C. Fries

**Affiliations:** 1 Department of Medicine, Division of Infectious Diseases, Stony Brook University, Stony Brook, New York, United States of America; 2 Department of Biomedical Sciences, Long Island University-Post, Brookville, New York, United States of America; 3 Universidad Francisco de Vitoria, Pozuelo de Alarcón, Madrid, Spain; 4 The Louis and Beatrice Laufer Center for Physical and Quantitative Biology, Stony Brook University, Stony Brook, New York, United States of America; 5 Department of Biomedical Engineering, Stony Brook University, Stony Brook, New York, United States of America; 6 Department of Molecular Genetics and Microbiology, Stony Brook University, Stony Brook, New York, United States of America; 7 Veterans Administration Medical Center, Northport, New York, United States of America; University of Toronto, CANADA

## Abstract

Similar to other yeasts, the human pathogen *Candida glabrata* ages when it undergoes asymmetric, finite cell divisions, which determines its replicative lifespan. We sought to investigate if and how aging changes resilience of *C*. *glabrata* populations in the host environment. Our data demonstrate that old *C*. *glabrata* are more resistant to hydrogen peroxide and neutrophil killing, whereas young cells adhere better to epithelial cell layers. Consequently, virulence of old compared to younger *C*. *glabrata* cells is enhanced in the *Galleria mellonella* infection model. Electron microscopy images of old *C*. *glabrata* cells indicate a marked increase in cell wall thickness. Comparison of transcriptomes of old and young *C*. *glabrata* cells reveals differential regulation of ergosterol and Hog pathway associated genes as well as adhesion proteins, and suggests that aging is accompanied by remodeling of the fungal cell wall. Biochemical analysis supports this conclusion as older cells exhibit a qualitatively different lipid composition, leading to the observed increased emergence of fluconazole resistance when grown in the presence of fluconazole selection pressure. Older *C*. *glabrata* cells accumulate during murine and human infection, which is statistically unlikely without very strong selection. Therefore, we tested the hypothesis that neutrophils constitute the predominant selection pressure *in vivo*. When we altered experimentally the selection pressure by antibody-mediated removal of neutrophils, we observed a significantly younger pathogen population in mice. Mathematical modeling confirmed that differential selection of older cells is sufficient to cause the observed demographic shift in the fungal population. Hence our data support the concept that pathogenesis is affected by the generational age distribution of the infecting *C*. *glabrata* population in a host. We conclude that replicative aging constitutes an emerging trait, which is selected by the host and may even play an unanticipated role in the transition from a commensal to a pathogen state.

## Author summary

This study shows that aging makes *Candida glabrata* more resilient and virulent *in vivo*. Aging results in a thicker cell wall that is also qualitatively different with respect to sterol composition, which conveys enhanced resistance to azoles and cellular stresses. We observed greater accumulation of old cells in competent mice, indicating that selection pressures in the host preferentially kill younger cells. Depletion experiments and mathematical modeling confirm that neutrophils represent the predominant selection pressure, and their removal dramatically alters selection pressures, resulting in a younger pathogen population. We propose that replicative age is an unanticipated virulence trait that is selected in the host environment and may play an important role in the switch from a commensal to a pathogen state.

## Introduction

*Candida glabrata* infections are common in immunocompromised patients and associated with prolonged treatment [1, 2], extended length of hospital stay, high costs and high mortality rates [3, 4]. Over the last decade, the incidence of *C*. *glabrata* infections has increased considerably due to higher numbers of immunocompromised patients, as well as broad empiric antifungal prophylaxis, which promotes colonization with azole-resistant *C*. *glabrata* [1]. *C*. *glabrata* is a very successful human pathogen because it has a high intrinsic stress tolerance, enabling it to withstand oxidative stress [5]. The yeast disseminates and attaches to host cells and indwelling devices, where it forms biofilms [6]. *In vitro* phagocytosed *C*. *glabrata* cells are able to survive and replicate inside human and murine macrophages [7, 8]. Imaging studies demonstrate that primary human neutrophils can kill or release phagocytosed *C*. *glabrata* [9]. Consequently, neutropenia constitutes a major risk factor for disseminated candidiasis in colonized patients [10, 11].

Most fungal infections are subacute or chronic [1, 10, 12]. A major barrier to improved antifungal drug therapy is a lack of understanding of how fungal populations change during chronic infection, and how this microevolution affects *in vivo* phenotypes and pathogenesis of the respective fungus [13, 14]. The pathogenic yeasts, *Cryptococcus neoformans*, *Candida albicans* and *C*. *glabrata* expand clonally in the host even though some of them retain the ability for mating and sexual reproduction [15]. Similar to other unicellular yeasts, *C*. *glabrata* is expected to undergo asymmetric mitotic divisions where original mother cells progressively age in a process referred to as replicative aging [16]. *Saccharomyces cerevisiae* has been the predominant model organism for investigating this process in yeasts. However, this fungus is rarely a pathogen in humans, and therefore research on it has historically focused on elucidating the molecular mechanisms that regulate aging [17].

Recent work in *C*. *neoformans* suggests that replicative aging can affect the pathogenesis of fungal populations [18, 19]. Specifically it was shown that old *C*. *neoformans* cells, referring to those with a higher replicative age, accumulate in spinal fluid during chronic infection, indicating the presence of a demographic shift within the infecting fungal population. Older cells manifested a thicker cell wall that rendered them more resistant to host response. This suggested that replicative age might be relevant in a clonally expanding *C*. *neoformans* population allowing it to gain resilience in the host environment. We proposed that older *C*. *neoformans* cells constitute the persister cells and contribute to the high treatment failure rate despite lack of antifungal resistance.

The intent of this study was to characterize the lifespan features of another important fungal pathogen, *C*. *glabrata*. Opposed to *C*. *neoformans*, this yeast can be a commensal, and it has been proposed that its resilience and high stress tolerance has evolved as a result of its intimate association with the mammalian host (for review [20]). The host is especially vulnerable for invasive disease, when the host becomes neutropenic and the major antifungal defenses are compromised. Then *C*. *glabrata* can shift from a commensal to an invasive pathogen. Therefore, we were particularly interested in studying the interplay between replicative aging of a *C*. *glabrata* population and the *in vivo* selection pressures due to neutrophil-mediated killing, which can be done by controlled depletion studies in the murine host. Our data demonstrate that aging leads to remodeling of the cell wall and that selection by neutrophils controls generational distribution within the *C*. *glabrata* population. We show that younger *C*. *glabrata* cells with more potential to adhere emerge in a neutropenic host, whereas older *C*. *glabrata* cells, which exhibit a thicker cell wall with an altered sterol composition mediate enhanced azole resistance and resilience and dominate in the immunocompetent host.

## Results

### Characterization of lifespan in clinical *C*. *glabrata* strains

**Fig 1:**
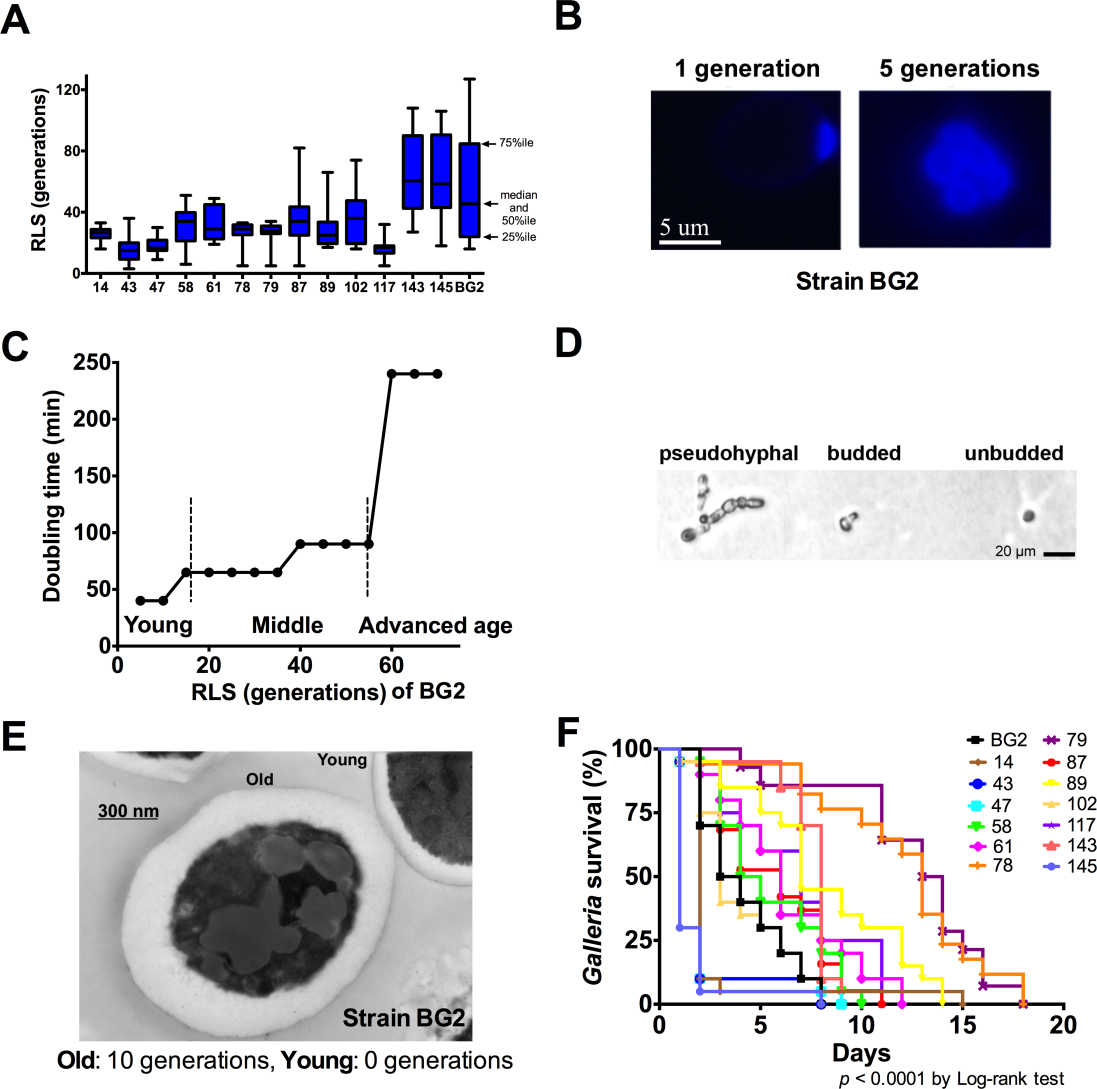
Phenotypic characterization of RLS in *C*. *glabrata*. (A) A box-and-whiskers plot demonstrates significant variability in RLSs of *C*. *glabrata* strains, shown as box plots with percentiles (n = 20 cells per experiment and 2–6 experiments compared by Log-Rank test). (B) Calcofluor stain reliably identifies generational age of *C*. *glabrata* (n = 100 cells). (C) Distinct doubling times during different lifespan phases, and (D) various cell morphologies were captured at time of death. (E) shows thicker cell wall in older BG2 cells by TEM (n = 100 cells compared by Student’s t-test). (F) *C*. *glabrata* demonstrated variable virulence in *G*. *mellonella* (n = 20 worms compared by Log-Rank test).

To investigate aging and its effects on various virulence-related phenotypes, we determined replicative lifespans (RLS) at 37°C on 14 clinical *C*. *glabrata* strains of different multilocus sequence types (MLST) and variable chronological lifespans (CLS) (Fig 1A and Table 1). The median RLS of *C*. *glabrata* strains varied from 15.0 (strain 43) to 61 generations (strain 143). Median RLS of cells across all strains was 29 generations and ranged from 3–127 generations, confirming the previous observation in *C*. *neoformans* [18, 19] that RLS is highly variable among strains, but a distinct and reproducible trait that defines the individual strain. High variability of RLS within a *C*. *glabrata* population was noted, where some cells exhibited a very short, and others a very long RLS. Neither prolonged nor shortened RLS was associated with a specific MLST type. In accordance with other publications [21], we found calcofluor reliably stains budscars, allowing us to identify the respective replicative age of older *C*. *glabrata* cells (Fig 1B and S1 Video). *C*. *glabrata* cells, regardless of their MLST, doubled every 45–50 min until they reached an advanced age when their doubling time slowed considerably from 90 to 240 min. Accordingly, the lifespan of *C*. *glabrata* strains could be divided into a young, middle, and advanced age phase (Fig 1C). Notably, at the end of their RLS, senescent *C*. *glabrata* cells arrested either as pseudohyphae, in a budded state, or as a single mother cell (unbudded) (Fig 1D). With every replication, the cell size and cell wall thickness increased (Fig 1E). Although considerable variability in relative cell body volume increase among strains was observed, there was no correlation between maximal volume and median RLS (Spearman r = -0.14, *p* = 0.78). Also despite variability, no correlation between virulence in the *Galleria mellonella* model and median RLS (Spearman r = -0.01, *p* = 0.96), or median CLS (Spearman r = -0.46, *p* = 0.10) was found. *C*. *glabrata* strains killed *Galleria* at markedly different rates (range 1–14 d, *p* < 0.0001) (Fig 1F). *C*. *glabrata* strains (14, 43, 47) that formed pseudohyphae were more virulent (median survival: 2 d).

**Table 1:** Characterization of clinical *C*. *glabrata* isolates.

Strain	MLST	Source of origin	Median CLS and range (days)
BG2	3	Vaginal	23 (20–25)
14	8	Urine	24 (22–25)
43	9	Urine	23 (20–25)
47	8	Urine	22 (20–24)
58	5	Urine	27 (25–30)
61	10	Urine	22 (20–24)
78	4	Blood	23 (20–25)
79	14	Blood	24 (22–25)
87	7	Urine	25 (23–27)
89	17	Urine	30 (27–33)
102	2	Urine	10 (7–13)
117	9	Urine	26 (22–24)
143	10	Urine	10 (7–13)
145	10	Urine	10 (7–13)

### Higher age makes *C*. *glabrata* more resilient to host attack and increases switching to more virulent variants

**Fig 2:**
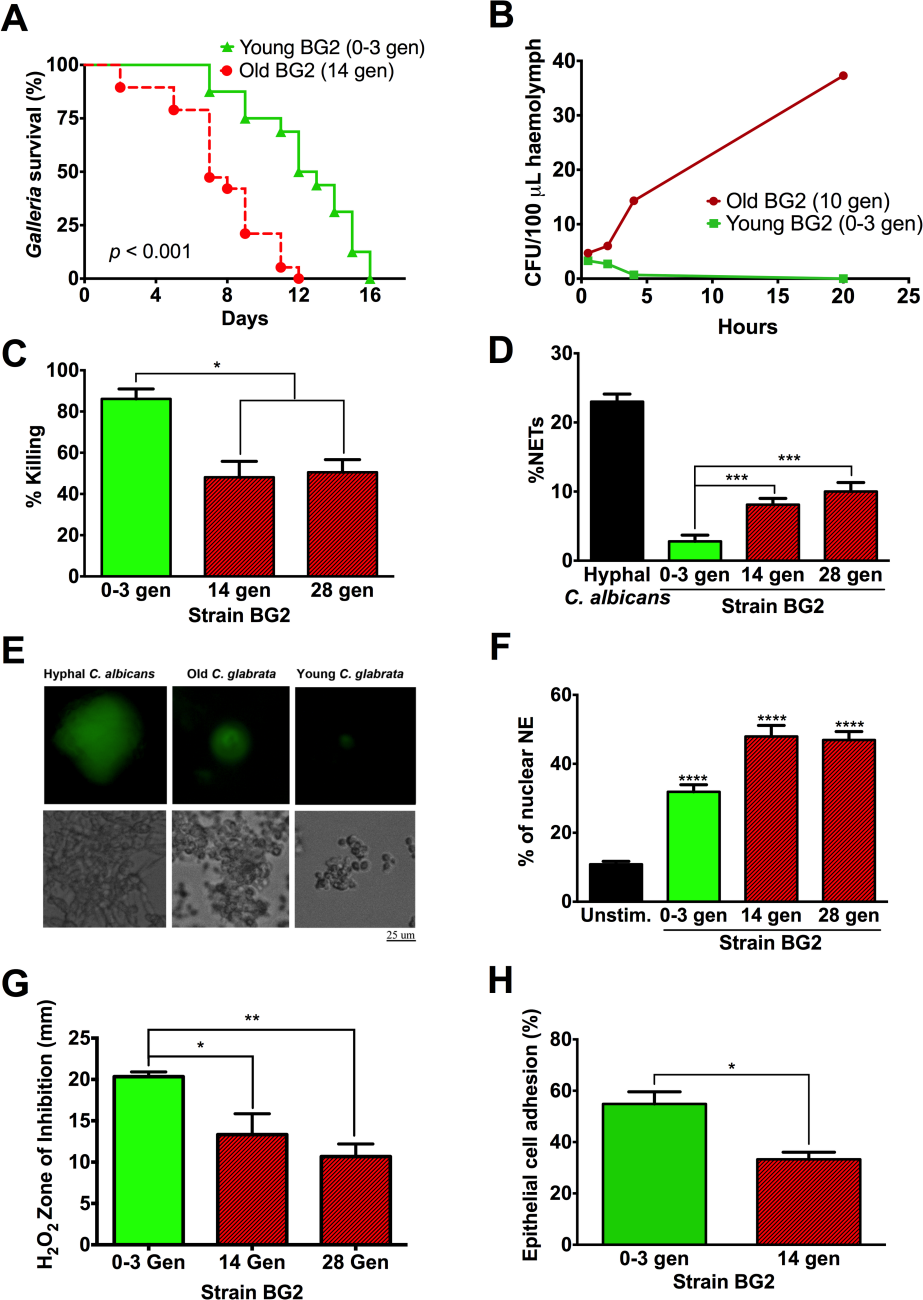
Enhanced resilience of older *C*. *glabrata* cells. (A) Older cells contributed to increased virulence in *Galleria* (n = 20 worms compared by Log-Rank test). (B) CFU counts continuously increased after *Galleria mellonella* infection with old BG2 cells (10 generations). Young BG2 cells (0–3 generations) were cleared before establishing infection within the first few hours post infection (n = 25 worms per time point). (C) Resistance to neutrophil-mediated killing was observed in older cells (experiments were run in duplicates, with six replicates, and compared by Student’s t-test). (D and E) NET induction was higher by old (14 and 28 generations) compared to young *C*. *glabrata* cells, but still significantly lower compared to that seen with hyphal *C*. *albicans*. % NETs indicates neutrophil nuclei >1,000 μm^2^ over total neutrophils, and upper panel in D shows sytox staining of nuclei (experiments were run in duplicates, with six replicates, and compared by Student’s t-test). (F) Neutrophil Elastase nuclear localization was higher in old (14 and 28 generations) compared to young *C*. *glabrata* cells and unstimulated neutrophils (10 neutrophils were analyzed per condition and compared by one-way ANOVA) (G) H_2_O_2_ disc diffusion assay shows smaller zone of inhibition for both 14 and 28 generation old cells (experiments were run in triplicates and compared by Student’s t-test). (H) Epithelial cell adhesion assays demonstrate decreased adhesion of older cells (experiments were run in triplicates and compared by Student’s t-test). *P < 0.05, **P < 0.01, ***P < 0.001, ****P < 0.0001.

**Fig 3:**
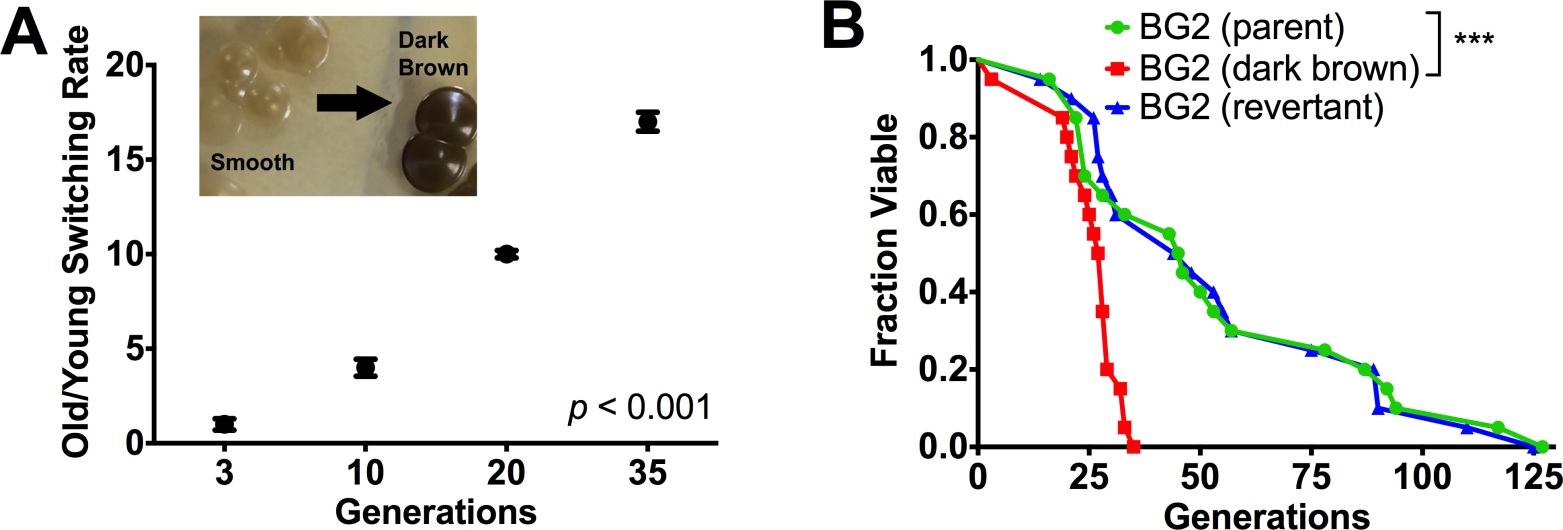
Switch variants have altered lifespan. (A) Phenotypic switching in BG2 from S to DB colony morphology (inset) increased consistently with aging to 17 fold (experiments were run in duplicates). (B) RLS of DB was shortened over 50% relative to S and reconstituted in the revertant colony (n = 20 cells over two experiments; DB v. S RLS compared by Wilcoxon Rank Sum Test). ***P < 0.001.

Similar to other yeast species, we hypothesized that replicative aging would augment *C*. *glabrata*’s resilience *in vivo* (Table 2). Old *C*. *glabrata* cells were isolated by biotin labeling a virgin population, allowing it ample time to divide and age, and then magnetically labeling it with streptavidin beads before sorting through a magnetic column. These cells, which henceforth we will label as “old,” are 10–14 generations, or for some experiments, 28 generations old. These are not young cells, but at this age are also far from senescence and thus have comparable growth dynamics to young cells (Fig 1C). The higher the replicative age of a cell, the rarer it becomes without stress: in a clonally expanding population a 14 generation old cell is rare and has a frequency of roughly 1:16,000 compared to other ages; this becomes 1:>10^8^ for a 28 generation old cell. We compared the virulence of these old cells to young ones of the standard laboratory strain BG2 using the *Galleria* model. These experiments demonstrated that old cells (14 generations, average diameter 6–8 μm) were significantly (*p* < 0.05) more virulent compared to young cells (0–3 generations, average diameter 4.1 μm) (Fig 2A). It was determined that younger cells were cleared quicker than older cells, while older cells tended to accumulate in the first few hours during infection (Fig 2B). Consistent with that finding, older cells were phagocytosed at a lower rate by *Galleria* haemocytes (S1A Fig) and human neutrophils (S1B Fig), and more resistant to neutrophil-mediated killing (Fig 2C). Although older *C*. *glabrata* cells induce more neutrophil extracellular traps (NETs) than young *C*. *glabrata* cells (Fig 2D–2F), relative to *C*. *albicans* hyphae the overall NET induction is considerably lower, and it is unclear if NETs truly kill these *C*. *glabrata* cells. Similar enhancement of virulence (S2A and S2B Fig), and resistance to neutrophil-mediated phagocytosis and killing (S2C and S2D Fig) was also documented in older cells of *C*. *glabrata* strains 89 and 117. Consistent with augmented resistance to neutrophil killing, older cells exposed to H_2_O_2_ exhibited smaller zones of no growth in a disc diffusion assay (Fig 2G and S2E Fig). Interestingly, older cells exhibited decreased adhesion to epithelial cells compared with younger cells (Fig 2H) suggesting that they would more successfully invade after dissemination. Lastly, the effect of aging on switching was investigated, in *C*. *glabrata*, where the phenotypic switch from the parental smooth (S) variant to the dark brown (DB) variant, is associated with enhanced persistence in mice ([22] and S2F Fig). These experiments demonstrate that replicative aging of BG2 cells from 2 to 35 generations consistently resulted in higher phenotypic switching rate (Fig 3A), a finding confirmed also in strain 89 (S2G Fig). Interestingly, in both strains, S to DB switching (Fig 3B and S2H Fig) resulted in over 50% loss of median RLS, which was reversible and regained in the revertant colony. These findings further support the conception that RLS is a highly regulated trait in a fungus.

**Table 2:** Summary of the phenotypic characteristics of 14-generation-old *C*. *glabrata* cells compared to young cells (strain BG2).

Phenotype	Effect in old cells
Cell volume	Increased (2.6 fold ± 1.0)
Cell wall thickness	Increased (2.3 fold ± 0.5)*
Doubling time	Increased (40 ± 10 min to 165 ± 75 min)*
Phagocytic killing	Decreased (32.3% ± 6.5%)**
NET induction	Increased (8.1% ± 1.0 from 2.7% ± 0.9)***
Adherence	Decreased (40.0% ± 8.5%)*
Hydrogen peroxide resistance	Increased (2.3 fold ± 0.3)*
Phenotypic switching rate	Increased (4–17 fold ± 0.4)***

*P < 0.05

**P < 0.01

***P < 0.001.

### *C*. *glabrata* resilience and enhanced resistance to antifungals is mediated by a cell wall remodeled in the process of aging

Given the above described striking phenotypes of resilience we pursued a transcriptome analysis comparing genes transcribed in young and 14-generation-old *C*. *glabrata* cells by RNA sequencing. Fungal cells were grown in YPD. RNAseq results were consistent with the observed distinct cell wall morphology on EM images of old cells and indicated that fundamental remodeling of the cell wall occurs throughout the process of aging. Specifically, several genes involved in ergosterol biosynthesis and sterol uptake (CAGL0I04246g, *ERG1*, *ERG3*, *ERG5*, *ERG6*, *ERG11*,) [23–27] were upregulated. *ERG19* and *ERG20* upstream in the “mevalonate pathway,” as well as CAGL0L09493g downstream in the “sito steryl glucoside synthesis” of ergosterol biosynthesis were also upregulated. Also CAGL0C04026g, a mannosyl transferase, which aids in protein glycosylation and uses farnesyl pyrophosphate, the endproduct of the mevalonate pathway was upregulated. In addition, several genes involved in cell wall remodeling (*KNH1*, *SUN4*) [28, 29], as well as the Hog pathway (*CDC42*, *STE20*, *MSB2*, CAGL0F03003g) [30–34] were significantly upregulated. Cell wall adhesins (*PWP3* and *PWP5*) [21, 35–38] and adhesion proteins (*AWP1*, CAGL0J02530g, CAGL0L06424g) [21, 36, 39] were significantly downregulated (virulence-associated genes are included in Table 3, complete list is available at GSE85985), which was predicted by our functional adhesion assays. Interestingly *EPA6*, a cell wall adhesin [40–45], and *FKS1*, a β1,3-glucan synthase [46, 47], both implicated in azole resistance were upregulated in old cells.

**Table 3:** List of genes upregulated in old *C*. *glabrata* cells known to be involved in antifungal resistance.

Gene Name	Gene ID	Fold Change	*P*-value	Function
	CAGL0I04246g	7.4	3.31E-16	Transcription factor involved in sterol uptake
*ERG11*	CAGL0E04334g	6.1	2.15E-17	Putative cytochrome P-450 lanosterol 14-alpha-demethylase
	CAGL0J07502g	6.0	2.97E-13	Putative protein similar to globins with a heme-binding domain
	CAGL0K04301g	5.6	2.99E-15	Mitochondrial Ser/Thr protein kinase
*TPO3*	CAGL0I10384g	5.3	8.12E-17	Predicted polyamine transporter of the major facilitator superfamily
*FKS1*	CAGL0G01034g	4.9	7.93E-14	Putative 1,3-beta-glucan synthase component
*KNH1*	CAGL0H07997g	4.7	2.29E-14	Involved in cell wall beta 1,6-glucan synthesis
*AQR1*	CAGL0J09944g	4.5	6.60E-15	Plasma membrane drug:H+ antiporter
*EPA6*	CAGL0C00110g	4.1	6.72E-13	Sub-telomerically encoded adhesin with a role in cell adhesion
*ERG3*	CAGL0F01793g	4.1	3.61E-16	Delta 5,6 sterol desaturase
	CAGL0M10219g	3.9	1.92E-13	Putative ceramide synthase component
	CAGL0M01870g	3.8	2.02E-12	Putative zinc finger protein
*SUR2*	CAGL0H01375g	3.7	7.68E-16	Predicted sphinganine hydroxylase with role in sphingolipid biosynthesis
*ERG5*	CAGL0M07656g	3.6	4.82E-16	Putative C22 sterol desaturase
	CAGL0F04917g	3.6	1.82E-13	Putative regulatory subunit for protein phosphatase
*YBT1*	CAGL0C03289g	3.3	6.12E-11	Putative ABC transporter involved in bile acid transport
*QDR2*	CAGL0G08624g	3.2	1.13E-15	Drug:H+ antiporter of the Major Facilitator Superfamily
*PFK1*	CAGL0F08041g	3.1	1.49E-13	Putative phosphofructokinase
*PDR16*	CAGL0J07436g	3.0	3.13E-11	Putative ABC transporter
*ERG6*	CAGL0H04653g	2.9	5.59E-15	C24 sterol methyltransferase
*RSB1*	CAGL0L10142g	2.2	2.36E-13	Sphingolipid flippase
*RTA1*	CAGL0K00715g	2.2	3.25E-13	Involved in 7-aminocholesterol resistance
*NCP1*	CAGL0D04114g	2.1	6.03E-13	Has NADPH-hemoprotein reductase activity

**Fig 4:**
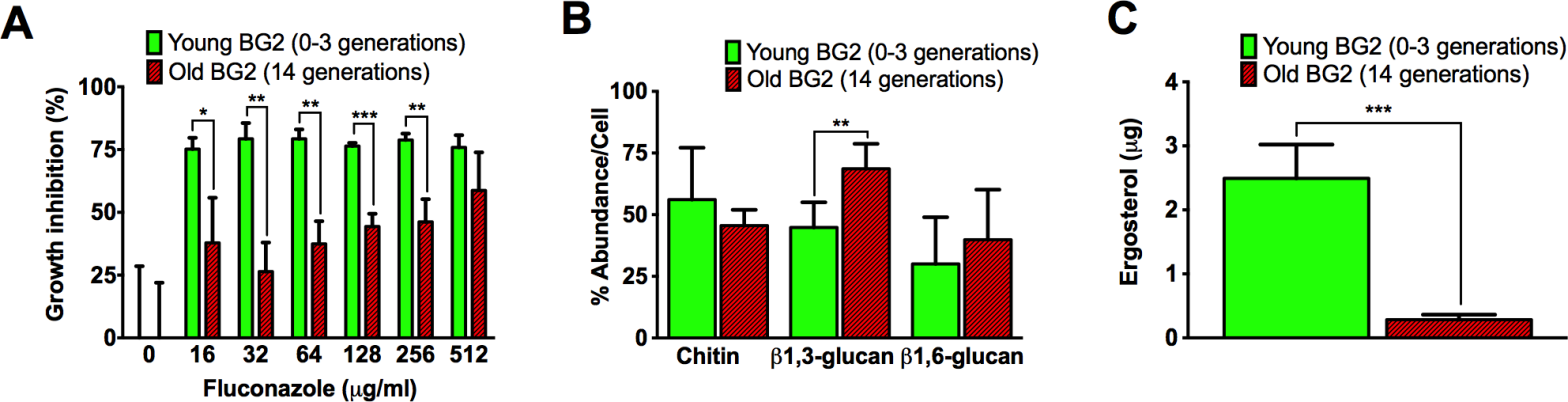
Enhanced resilience of older *C*. *glabrata* cells stems from cell wall remodeling. (A) *C*. *glabrata* cells aged under sub-therapeutic fluconazole were more resistant to growth inhibition than younger cells when subjected to various concentrations of fluconazole for 4 h (experiments were run in triplicates and compared by Student’s t-test). (B) Older *C*. *glabrata* cells contain a slightly higher quantity of β1,3-glucans, but comparable amounts of β1,6-glucans and chitin (experiments were run in triplicates and compared by Student’s t-test). (C) Lipid analysis by GC-MS indicated that younger *C*. *glabrata* cells contain a higher amount of ergosterol compared to 14-generation-old cells (experiment was run in triplicates). *P < 0.05, **P < 0.01, ***P < 0.001.

Given these results we predicted that old cells would be more resilient under the selection pressure of azole treatment. Older (5–7 generations) and young (0–2 generations) *C*. *glabrata* cells were grown and aged for 15 h under sub-therapeutic levels of fluconazole (32 μg/mL), and then subjected 4 h to increasing fluconazole concentrations (0–512 μg/mL) before plating on YPD plates. At fluconazole concentrations of 16–256 μg/mL, the younger cells were inhibited/killed at significantly higher rates than the older cells (Fig 4A). Specifically, failure of young cells to grow on fluconazole containing plates (16 to 64 μg/mL) ranged from 75–79%, respectively. Whereas failure of old cells to grow ranged from 26% (32 μg/mL) to 59% (512 μg/mL). Inhibition was relatively stable across various fluconazole levels for the young cells, whereas growth inhibition of older cells tended to slightly increase as the amount of fluconazole increased. These data suggest that older cells grown under selection pressure of sub-therapeutic fluconazole are significantly more resistant to subsequent fluconazole exposure compared to young cells exposed to the same fluconazole concentrations.

Transcriptome analysis (GSE85985) identified upregulation of several β1,3-glucan enzymes: a synthase (*FKS1*) that is functionally redundant with Fks2, and in which “hot spot” mutations confer resistance [48], a exo-1,3,beta-glucosidase (CAGL0G09515g), a transglycosidase (*GAS5*) with an elongation role [21], and an ortholog with osmosensor and biosynthetic activity (CAGL0F03003g). Also, *KNH1* [28], which is involved in 1,6-glucan synthesis, was upregulated, while a 1,4-alpha-glucosidase (CAGL0G02717g) was downregulated. In addition to the glucan signature, CHS3B, a Class IV chitin synthase that has a calcineurin pathway- and Tor1p-dependent role in cell wall integrity [49, 50] was upregulated. Nevertheless, major quantitative or qualitative differences for content of chitin, β1,3-, and β1,6-glucans could not be established by employed biochemical methods. Old *C*. *glabrata* cell walls contained both glucans, and only a slightly higher quantity of β1,3-glucans was noted, whereas chitin and β1,6-glucan content did not differ (Fig 4B).

Detailed transcriptome (Table 3) and gene ontology analysis (Table 4) identified enrichment of steroid metabolic and biosynthetic processes. Therefore, we analyzed the lipid composition of older *C*. *glabrata* cells to investigate if the upregulation of the ergosterol pathway leads to accumulation of sterol intermediates. The ergosterol content of old and young cells was quantified by GC-MS and normalized both to cell number as well as to lipid content. This quantitative analysis demonstrated approximately 10 fold lower ergosterol levels in old compared to young cells (Fig 4C), whereas analysis of the downstream sito steryl glucosides revealed slightly increased accumulation (10.5 vs 6.6 μg/ml). This latter quantification was done by LC-MS in pooled samples of 14-generation-old compared to young cells (0–3 generations old).

**Table 4:** Gene ontology enrichment analysis for genes upregulated in old *C*. *glabrata* cells.

Gene ID	*P*-value
nucleobase transport	8.35E-07
organic hydroxy compound metabolic process	0.0008
rRNA processing	0.00228
thiamine-containing compound metabolic process	0.00286
thiamine metabolic process	0.00286
steroid metabolic process	0.00349
rRNA metabolic process	0.00369
organic hydroxy compound biosynthetic process	0.00432
sterol biosynthetic process	0.00713
sterol biosynthetic process	0.01031
sterol metabolic process	0.01167
alcohol metabolic process	0.01393
alcohol biosynthetic process	0.01873
maturation of SSU-rRNA from tricistronic rRNA transcript (SSU-rRNA, 5.8S rRNA, LSU-rRNA)	0.01907
thiamine-containing compound biosynthetic process	0.03803
thiamine biosynthetic process	0.03803
ribosome biogenesis	0.04021
ribosomal small subunit biogenesis	0.04025
RNA phosphodiester bond hydrolysis, endonucleolytic	0.04409
ribosomal large subunit biogenesis	0.04441
cellular alcohol metabolic process	0.04981

Taken together, the transcriptome data show that cell wall remodeling genes, several of which are already implicated in *C*. *glabrata* virulence and antifungal resistance are differentially regulated in older cells. Older cells exhibit markedly upregulated ergosterol pathway associated genes and greater resistance under increasing fluconazole selection. Furthermore, biochemical analysis of older *C*. *glabrata* cells show differences in ergosterol content and cell wall composition.

### Host neutrophils select older cells *in vivo*

**Fig 5:**
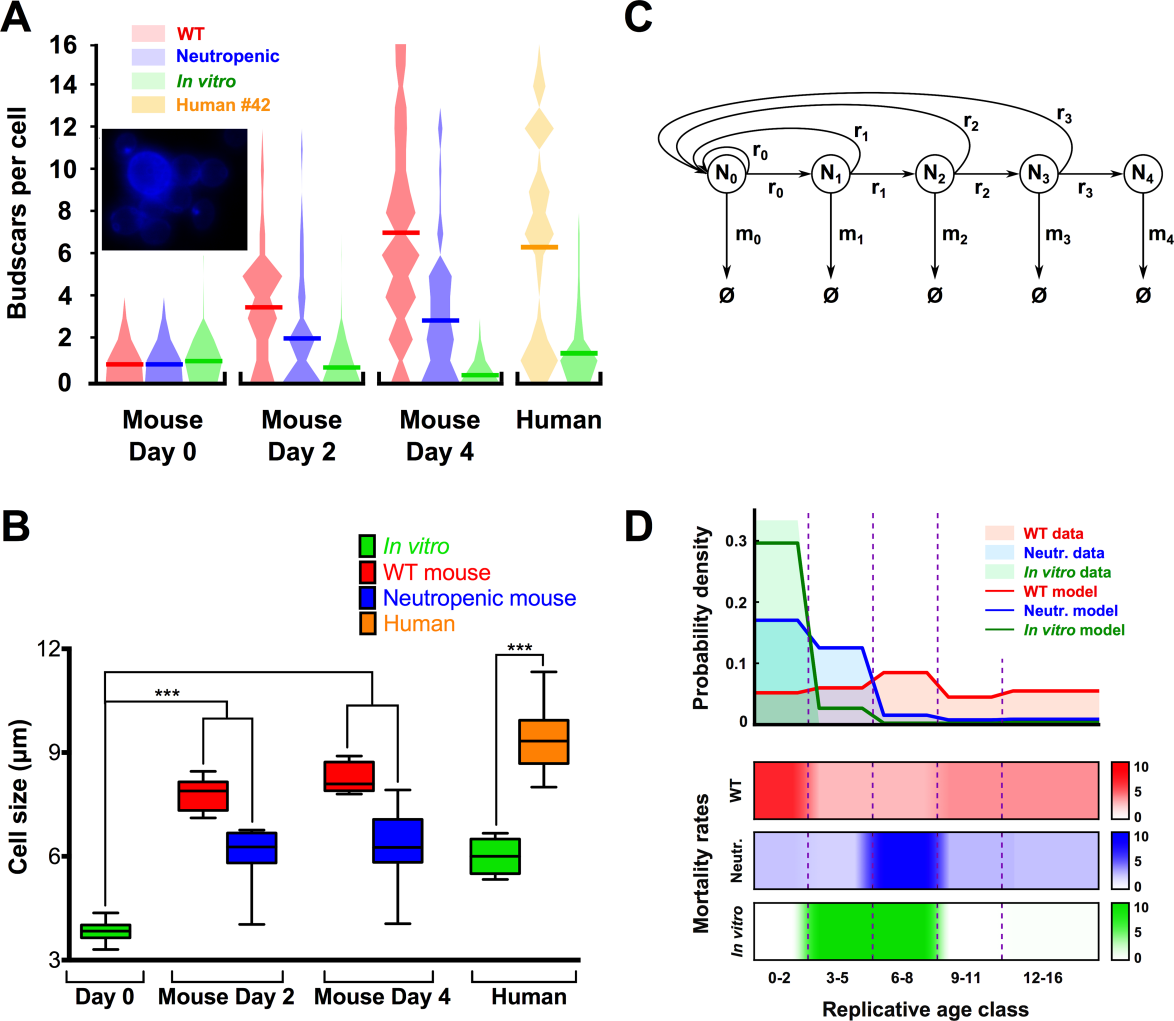
Older *C*. *glabrata* cells accumulate *in vivo*. (A) Calcofluor stain shows BG2 cells with increased budscars in mouse kidneys (inset). Significantly more BG2 cells with high budscar counts (mean budscars represented by line through plot) were found at day 2 and 4 in kidneys of WT compared to neutropenic mice injected intravenously, and also compared to day 0 in either host. Higher budscar counts were also found in *C*. *glabrata* from candiduric patient when compared to the same strain grown *in vitro* (#42). (B) BG2 cells were larger at days 2 and 4 in kidneys of WT mice compared to neutropenic mice and inoculum (day 0). (C) Model of expansion or contraction of a fungal cell population with five age classes: 0–2, 3–5, 6–8, 9–11 and 12–16 replications, whose populations are given by N_0_, N_1_, N_2_, N_3_ and N_4_, respectively. *r*_*0*_…*r*_*3*_ are replication rates, and *m*_*0*_…*m*_*4*_ are mortality rates of each age class. (D) Theoretical model can reproduce experimental results and confirm differential selection hypothesis. Upper panel: Probability distributions of replicative ages found experimentally (shaded areas) overlaid with results from the model parametrized with the most optimal parameters found using optimization (lines). Lower panel: Mortality rate profiles that yielded the best fits of the model to the data for each condition. Precise mortality rates were *m* = [6.9 3.0 2.8 4.1 4.0] (WT host); *m* = [2.8 2.3 9.3 2.9 2.8] (neutropenic host); *m* = [0.0, 10.4, 10.4, 0.0, 0.5] (*in vitro*).

In a clonally expanding pathogen population, old cells statistically cannot dominate within the population unless forceful selection pressures are operative to promote their emergence and kill off younger cells. Based on the aforementioned *in vitro* assays, which suggest that old cells have a biological advantage under certain selection pressures, we hypothesized that the immune response of the host controls the generational distribution of a pathogen population by differential mortality. In support of this, larger *C*. *glabrata* cells with high budscar count were observed in human patients (n = 6) with urinary tract infections. Those patients were not neutropenic, and violin plots of the budscar counts indicate higher accumulation of old cells from *in vivo* derived yeast cells when compared to the same cells grown up without selection pressure with *in vitro* cultures (Fig 5A shows data from patient #42, and S3A Fig shows data from the remaining patients). Disseminated *C*. *glabrata* infections commonly occur in neutropenic patients, and thus we predicted that neutrophils constitute the selective force in mice, as they are the key effector cells that contain *C*. *glabrata* infection [51]. To test this hypothesis *in vivo*, the effect of neutrophil depletion was examined on the generational distribution of an infecting *C*. *glabrata* population with different strains (BG2 and 89) injected intravenously into wildtype (WT) or neutropenic mice. The latter immune status was achieved by injecting antibody RB6-8C5, which transiently depletes neutrophils in the mouse. Subsequently, *C*. *glabrata* was harvested from homogenized kidneys every 2 days, and yeast cells were directly stained with calcofluor to assess budscar count as a measure of replicative age. In addition, cell size was measured as increase in size correlates with older age. The violin plots demonstrate shift in generational distribution. Specifically, a significantly higher percentage of older cells, as demonstrated by increased budscars and cell size were found in WT mice when compared to neutropenic mice for both strains BG2 (Fig 5A and 5B, and S1 Table) and 89 (S3B Fig, and S1 Table). This supported the hypothesis that old cells are selected due to increased killing of cells by host neutrophils. Consequently, in neutropenic mice the pathogen population is younger, since there were fewer neutrophils to kill them, and the generational distribution was more similar to a population that had grown *in vitro* without exposure to selection forces.

### Computational modeling supports differential selection hypothesis

To confirm the experimental results theoretically and gain additional insights, the selection pressure on cells of various ages was determined based on the distribution of replicative ages from the mouse experiment (Fig 5A). A system of ordinary differential equations was developed representing the time-dependent population sizes of five age classes (S4 Fig) to evaluate the age distributions resulting from various mortality rates on each age class (Fig 5C). Many initial mortality profiles (sets of mortality rates) were systematically explored, then local optimization was used to vary each mortality rate separately (S5 Fig). This returned the corresponding best-fit age distribution given the initial profile. Chosen from all local optima, the global-optimum mortality profile was robust to the initial profile, with the local optimizer converging on it for 38%, 52% and 63% (out of 3125 initial profiles) of the search space for WT, neutropenic, and *in vitro* populations, respectively; S6 Fig).

The experimental age distribution superimposed with the results from the model were parametrized with the most optimal mortality rates, as given by the global optimization (Fig 5D). The model fit the experimental age distribution closely for all three experimental populations. It was also able to capture the overall dynamics of the *C*. *glabrata* infections *in vivo* (S4 and S5 Figs). The estimated mortality rates took different forms for each condition (Fig 5D): high on the first age class and lower on the rest for WT mice; high on the middle age class and lower on the rest for neutropenic mice; very low on all age classes except the second and third for the *in vitro* population. Comparing the mortality rates with each other, the mortality profile of neutropenic mice was similar to that of *in vitro* cultures (Pearson r = 0.54, *p* = 0.35). Interestingly, mortalities in WT mice showed an opposite pattern; they were highest for young cells (r = -0.41, *p* = 0.49 between WT and neutropenic; r = -0.71, *p* = 0.18 between WT and *in vitro*). The lack of significance was a result of the comparison between two sets of five values (the number of age classes).

Overall, the computational results showed that in order to generate the replicative age distributions observed experimentally, a competent host must cause selection pressures on each age class that are different from those of a neutropenic host (as well as an *in vitro* population). This served as further confirmation of the hypothesis that neutrophils constitute the main selection pressure in a competent host, since the mortality profile with respect to age of a neutropenic host was more similar to that of an *in vitro* population than a competent host.

## Discussion

To our knowledge, this is the first study to investigate replicative aging in clinical *C*. *glabrata* populations. Our data link replicative aging to pathogenesis in this important fungal pathogen. Specifically, we demonstrate that the natural process of aging results in a remodeling of the fungal cell wall leading to enhanced resilience. Furthermore, we show that neutrophil-mediated killing comprises the host’s selection pressure, which shifts the generational distribution of a *C*. *glabrata* population *in vivo*. Thus, this study supports a conceptually novel understanding of pathogenesis, where replicative aging in a yeast population facilitates demographic changes in the presence of a selective host response. Hence, the natural process of replication can be exploited as a unique mechanism of adaptation that contributes to phenotypic variation, resilience, and virulence. Since the phenotype of aging is not genetically inherited, but only seen through shifting selection pressures that promote persistence of older cells, the high risk of random permanent mutations is avoided. Instead constant rejuvenation through budding provides an assurance that all adaptive changes can be rapidly reversed as the daughter inherits asymmetrically [52, 53] and allows the emergence of a new generation.

Our original study indicated accumulation of old *C*. *neoformans* cells in chronic cryptococcosis [19, 54]. That work led to the hypothesis that aging is associated with pathogenesis and that the high number of *C*. *neoformans* cells with decreased remaining RLS, which we recovered from spinal fluid of infected rats or humans, represent old cells, which are statistically unlikely to occur in a clonally expanding population unless selected. To examine this hypothesis further and in depth, we chose *C*. *glabrata*, a commensal that can become pathogenic to cause invasive disease predominantly in neutropenic patients. Using this yeast we could study directly the association of aging and fungal pathogenesis, because opposed to *C*. *neoformans*, it maintains budscars on its cell surface when it continues to replicate, and therefore the actual generational age can be determined in real time. In addition, neutrophils, which are key host defense cells in several human and murine *Candida spp*. infections [12, 51, 55], can be studied *in vitro* and depleted *in vivo*. However, given the lack of prior studies on replicative aging in *C*. *glabrata*, baseline assumptions about replicative aging had to first be validated for this species.

We found considerable variability of RLS among clinical *C*. *glabrata* strains similar to that observed in other fungi [18, 19, 56, 57]. *C*. *glabrata* cell size increased consistently with each replication. In contrast to *S*. *cerevisiae* [58], maximal cell volume did not predict RLS, and future imaging studies will need to clarify whether time to first bud emergence correlates with length of RLS as recently demonstrated for *S*. *cerevisiae* [59]. Variable length of exposure to the intracellular environment of phagocytic cells has been associated with change in the virulence of several *Candida* spp. [13, 14]. Thus, it is conceivable that the intracellular environment of other phagocytic cells, especially macrophages, also contributes to variability in replicative age among the clinical strains.

*C*. *glabrata* can colonize the human host, and cause disease in humans only when host defenses fail and unchecked growth of the *C*. *glabrata* population ensues. We were therefore most interested in comparing resilience-associated phenotypes in young and old cells. Consequently, *in vivo* resilience of old and young *C*. *glabrata* cells was compared using a well-established infection model of *G*. *mellonella* [60–62]. These data demonstrate that aging indeed enhances resilience, and consequently virulence of this pathogen. Specifically, we found that older cells of both short and long-lived strains killed *Galleria* significantly faster than younger cells of the same *C*. *glabrata* strain. It is important to emphasize that 14-generation-old cells are far from senescence, and therefore fitness would not be significantly decreased. Accordingly, we show that 14-generation-old cells exhibit comparable doubling rates. Several important phenotypic differences between young and old cells explain the observed resilience of the older cells.

We demonstrated resistance of older cells to killing and phagocytic uptake. Young cells do not evade the basic immune response of *Galleria*, and they are therefore killed more rapidly and cannot establish infection as rapidly as the older *C*. *glabrata* cells. *Galleria* has an innate immune system composed of six types of phagocytic cells [60]. Consistent with our finding is the observation that larger *C*. *neoformans* cells are also more resistant to uptake by *Galleria*’s phagocytic cells [63].

Furthermore, our data shows that age confers resistance to intracellular killing after phagocytic uptake. In mammals, one contributing facilitator of killing in neutrophils are reactive oxygen species [55, 64]. Although *C*. *glabrata* is closely related to *S*. *cerevisiae*, higher resistance to ROS has evolved in this yeast as an adaptation to the intracellular environment of phagocytic cells, enabling the fungus to persist in the host [5]. Our data now indicate that aging augments this resistance, explaining the resistance of older *C*. *glabrata* cells to neutrophil-mediated killing. Transcriptome data indicate that genes in the Hog pathway, including *CDC42*, *STE20*, *MSB2*, were significantly upregulated in older cells, and this pathway is a well-known regulator of cell wall damage imparted by various stresses [65].

Hence, in order to be protected, older cells make a thicker cell wall through a concerted mechanism involving gene regulation, protein reorganization, and lipid remodeling. The observation that glucan is much more abundant in old cells compared to young cells is supported by recent studies in *Candida tropicalis*, where a thicker cell wall rendered strains less susceptible to amphotericin B and more resistant to stress responses [66]. Also in this instance, the authors found more beta-glucans in strains with thicker cell wall with no change in chitin content, similar to our findings. Our studies extend these observations to another fungus. We also documented a significant decrease in ergosterol in older cells, which could lead to a re-organization of the plasma membrane. In addition, sito steryl glucosides, a downstream product of ergosterol synthesis is known to accumulate in the plasma membrane [67, 68]. Data from *Saccharomyces* indicate that mutations of the ergosterol pathway affect the cell wall integrity [67]. Furthermore, vesicular trafficking and release of extracellular vesicles may be affected by ergosterol level, which may also modulate the immune response [69]. The increased azole resistance could be attributed to increased expression of the azole target, Erg11p. It is noteworthy that although *ERG11* and several other ergosterol pathway genes are markedly upregulated in old cells, ergosterol levels were found to be significantly lower in old cells. Several factors could contribute to this finding. First the overexpression of upstream mevalonate pathway genes, *ERG19* and *ERG20*, may redirect the sterol intermediates to the side pathway; as such we found a mannosyl transferase (CAGL0C04026g) upregulated. This enzyme aids in protein glycosylation [70]. Second, the accumulation of sito steryl glucosidase, which is downstream of ergosterol may lower the ergosterol levels. This may also promote overexpression of increased uptake. Future studies examining the azole influx and efflux in young and old cells may shed more light on how azole resistance is mediated [67]. Third, the transcriptional upregulation in older cells may be due to a compensatory response to ergosterol depletion. If older cells have mutations in the enzymes involved in ergosterol biosynthesis, it may explain the low ergosterol level and the resistance to fluconazole. Further studies are clearly needed to test these possibilities.

The cell wall constitutes the key defense against all external attacks. In addition it is also recognized by the host immune system, which can make both effective and ineffective harmful immune responses as a result of host-pathogen interaction [71, 72]. Lastly because cell walls of fungi are fundamentally different from the bi-lipid membranes of human cells, they are very attractive targets for antifungal therapy. The concept that cell walls are dynamic and that structural realignment occurs as a result of stress induced upregulation of signaling pathways is not novel and has been described for other yeast as well [73–75]. In our case, however, cell wall reorganization is not a response to an environmental change per se, but a process that is merely driven by an internal clock, namely the natural process of aging. This process is controlled by evolutionarily conserved signaling pathways similar to those of environmentally induced stress (Hog1), cell integrity (Mkc1, Cek1) and ergosterol metabolism (Erg11, Erg3, Erg5, Erg6, Sur2) [76].

A less obvious mechanism of how aging can affect pathogenesis is by augmenting phenotypic switching rates to more persistent or virulent variants. Older *C*. *glabrata* cells switch at a significantly higher rate to dark brown colony variants, which colonize the host more efficiently [22]. Interestingly, both the hypervirulent mucoid (*C*. *neoformans*) [19], and DB (*C*. *glabrata*) switch variants exhibit more than 30 generations shortened median RLS, a loss which is instantly recovered with reversal to the parent phenotype. This finding further supports the notion that isogenic strains with shortened RLS may be more virulent because of rapid aging as the more resilient “old age-associated” phenotypes are acquired earlier. In addition, it underscores the fact that lifespan, although variable among *C*. *glabrata* strains, is a highly regulated trait in the individual pathogenic yeast cell.

Lastly, it should be emphasized that staying young also has some benefits. Our results suggest that adherence to epithelial cells is enhanced in young cells. Adherence of *C*. *glabrata* to host cells is mediated in part by the Epa family of adhesins, which are encoded largely at subtelomeric loci and subject to transcriptional silencing of *SIR* genes [77]. *EPA6* for instance is derepressed in response to limiting levels of nicotinamide adenine dinucleotide, which results in reduction of Sir2-mediated histone deacetylation and loss of silencing [41]. In *S*. *cerevisiae*, Sir2 levels decrease in old cells, and therefore one would predict higher adherence as a result of derepressed *EPA* expression [78]. However, it is conceivable that the thicker cell wall of older *C*. *glabrata* cells may affect surface exposure of Epa proteins, some of which reside in the *C*. *glabrata* cell wall [79]. Nonetheless in *C*. *glabrata*, adhesion induces actin-mediated phagocytosis and an increased inflammatory cytokine response [6]. Thus, the decreased adherence observed in the older cells in our studies is consistent with their decreased phagocytosis and ultimately decreased killing.

In *C*. *glabrata*, stainable budscars permitted us to assess in real-time the generational age distribution of an *in vivo* expanding *C*. *glabrata* population. This allowed us to design experiments that directly assessed the effect of host response on the emergence of old cells. First, we demonstrate by directly counting budscars that the *in vivo* derived *C*. *glabrata* population includes a large percentage of cells with many budscars, the hallmark of advanced generational age. Importantly this observation was confirmed with *C*. *glabrata* cells derived from patients with candiduria. Next, our hypothesis that the shift to an older population is the result of selection by a host response was tested by removing the selective force through depletion of neutrophils in mice. These cells were chosen as they represent the key inflammatory cells that contain the infection *in vivo* [51]. The results support our hypothesis and show that depletion of neutrophils shifts the age distribution of the pathogen population towards a younger population similar to that seen in exponential *in vitro* growth of *C*. *glabrata*. One concern is that budscar analysis maybe biased because the younger cells adhere better than the older cells. This is, however, unlikely as we homogenized the cells thoroughly and lysed away the murine tissue cells to achieve a single cell population for budscar staining. In addition, adhesion to epithelial cells would also be expected in neutropenic mice. Another concern is that budscar measurement of very old cells (30 generations) has not been experimentally confirmed. Although this concern may be valid, it would underestimate the age of old cells, and the result would be even more striking.

Further, by constructing and directly manipulating a mathematical model of an expanding or contracting cell population, we were able to generate replicative age distributions that matched our experimental data. This tells us that the different age distributions obtained *in vivo* were a direct result of differential selection (mortality) on each age class. This further supports our hypothesis that effective killing of young cells is the driving force behind the demographic shift in the fungal population derived from the host, and therefore that neutrophils are the cause of the shift. Future studies should aim to correlate the differential responses of neutrophils observed in our *in vitro* studies with the intracellular or extracellular locations of fungi *in vivo*. This might shed better insight into this particular selective force.

Conventional wisdom imparts that stress promotes aging [80]. Ironically, here we show that aging promotes stress resistance in the pathogen, *C*. *glabrata*. The most stressful environment for a commensal and a pathogen is the host, and we show how loss of host response results in a younger pathogen population. By contrast, in a competent host the younger cells are killed and the older yeast cells persist. Thus, while aging endows *C*. *glabrata* strains with increased resilience and promotes commensalism, youth selected in the neutropenic host may convey fitness, namely the ability to disseminate and adhere, and thus promote the pathogen state. Given that aging is a regulated process, it could potentially be targeted for antifungal drug development, which is in desperate need of new targets [81, 82]. It is conceivable that combination of antifungals and lifespan-altering drugs would result in synergy if they were tested in the host environment. We propose that replicative aging represents a virulence-associated trait of eukaryotic pathogen populations that contributes to microevolution of pathogens in the host. Importantly, this mechanism of microevolution may be relevant for other eukaryotic pathogens that either cause chronic infections, or alter between the commensal and pathogenic lifestyle.

## Methods

### Ethics statement

All animal experiments were carried out with the approval of the Stony Brook University Animal Institute. The protocol #628261 was approved by the Institutional Animal Care and Use Committee at Stony Brook. The study in strict accordance with federal, state, local and institutional guidelines that include “The Guide for the Care and Use of Laboratory Animals,” “The Animal Welfare Act,” and “Public Health Service Policy on Human Care and Use of Laboratory Animals.” *Candida* from patient urine samples was obtained with the approval of the Stony Brook Institutional Review Board (protocol #648612). No consent was required as the data was de-identified and anonymous. Neutrophils were isolated from donor blood (New York Blood Center) with the approval of the Stony Brook Institutional Review Board (protocol #646298).

### *C*. *glabrata* strains

Clinical *C*. *glabrata* strains from the United States (Table 1) stored at -80°C were streaked for single colonies on Yeast Peptone Dextrose agar (Difco) at 37°C thrice before analysis. Multilocus Sequence Typing of *C*. *glabrata* strains was performed with primers as previously described [83].

### Replicative and chronological lifespan

RLS was determined for *C*. *glabrata* strains by adaptation of *S*. *cerevisiae* methods [16]. Briefly, new buds from a virgin cell (n = 20) on YPD agar were separated at the end of each division using a 25 μm needle (CoraStyles) on a tetrad dissection microscope (Zeiss) at 250X magnification. Plates were returned to 37°C at the end of each division, and cells that had not divided for 24 h were declared dead. CLS was determined by adaptation of *S*. *cerevisiae* methods [17]. Briefly, cells were grown for 3 d in YPD broth (Difco) until they reached stationary phase, then transferred to dH_2_O, and their viability was determined by plating colony forming units (CFU) every 2 d until 99% of the cells were dead.

### Isolation of *C*. *glabrata* cells with advanced replicative age

Counter-flow centrifugal elutriation was used to isolate old cells as previously described [18]. In the methods below, young describes cells that gave rise to fewer than 3 daughters, and old describes cells that are a third or more into their lifespan (approximately 14 generations old) or more (28 generations old) unless otherwise stated. With this method, the flow and/or the centrifugation speed can be adjusted to collect only those cells whose sedimentation velocity (the rate at which molecules move in response to the centrifugal force generated in a centrifuge) falls in a particular range. To avoid clumping, cells were washed twice with 30 mM EDTA, and then thrice with PBS. Briefly, newly budded *C*. *glabrata* cells were isolated by elutriation (Beckman JE-5.0 rotor in a Beckman J-6B centrifuge; Beckman Instruments Inc.), and labeled with Sulfo-NHS-LC-Biotin (Thermo Scientific). The newly budded and labeled cells were grown for several generations, and collected by first binding them to streptavidin-conjugated magnetic microbeads (Miltenyi Biotec), then isolating them on a magnetic column (Miltenyi Biotec). Unbound young yeast cells (0–3 generations old) that washed off the column and had been exposed to similar manipulations were used as controls. Purity of old cells was confirmed by Fluorescein isothiocyanate (FITC)-staining of the streptavidin-labeled cells.

### Imaging

Cells suspended on YPD agar were imaged at 100X magnification on an Olympus AX70 microscope, and cell size was measured in Adobe Photoshop CS6 for Macintosh. For budscar stains, old *C*. *glabrata* cells were isolated as described earlier, or specimens from murine or human infection were directly stained with 100 μg/ml Calcofluor (Sigma) in dH_2_O for 5 min at room temperature in the dark, followed by washes in dH_2_O. Images were taken at 1000X magnification under oil immersion in the blue channel with a Qimaging Retiga 1300 digital camera and processed in the Qcapture Suite V2.46 software (Qimaging). Transmission electron microscopy was performed with the help of Stony Brook University microscopy facility. Briefly, virgin and 10-generation-old, and *C*. *glabrata* cells were harvested, resuspended in 5% glutaraldehyde, and fixed for 2 h at room temperature. Samples were postfixed in 1% OsO_4_ at 4 ^o^C, and after several washing and dehydration steps, embedded in araldite. Ultrathin sections (70–74 nm) were cut using an ultramicrotome (Leica EM UC7), contrasted with lead citrate, and imaged using a JEOL JEM 1400 TEM.

### Neutrophil-mediated killing and neutrophil extracellular trap assay

Neutrophils were isolated from donor blood (New York Blood Center) as previously described [84]. Neutrophil-mediated killing was determined on young or old *C*. *glabrata* cells as described [85]. Briefly, neutrophils were diluted in RPMI 1640 medium with 1% human sera for opsonization and allowed to adhere to 96 well plates for 30 min at 37°C and 5% CO_2_. *C*. *glabrata* cells were isolated as described earlier and added in a fungus-to-neutrophil ratio of 1:10 or 1:100. Wells with no neutrophils were included as controls. The plate was incubated for 30 min to allow phagocytosis, then incubated an additional hour to allow killing. Appropriate dilutions were plated on YPD plates and incubated at 37°C for 48 h. All experiments were performed in six replicates, and the killing percentage was calculated by comparison of CFU 1 h after phagocytosis. NET production and Nuclear Elastase (NE) were measured as previously described [86]. Briefly, for NET production, 5 x 10^4^ neutrophils/ml were plated on 24 well plates in Hank’s balanced salt solution with 3% sera, and *C*. *glabrata* yeast (young or old), or preformed *C*. *albicans* hyphae were added at an MOI of 10. After 4 h of incubation, NETs were visualized by the addition of SYTOX (S702, Invitrogen), and imaged at 40x magnification in at least six random fields. The mean percentage and standard deviation of neutrophil nuclei larger than 1,000 μm^2^ over the total neutrophils was calculated. NE nuclear co-localization was determined after 5 x 10^4^ neutrophils were plated on glass coverslips 1 h prior to stimulation. After 4 h of stimulation, cells were fixed with 2% paraformaldehyde for 20 min at 37°C. After immunofluorescence staining was performed with anti-neutrophil elastase (Abcam) and DAPI, confocal z-series (every 0.8 mm) covering the whole neutrophils were used to quantify total NE and nuclear localization using ImageJ. A mask of the DAPI channel was created to measure the NE nuclear localization, and the total area and percentage of co-localization in individual channels was used to yield the total NE signal (10 neutrophils per condition were used).

### H_2_O_2_ disc diffusion

Young or old *C*. *glabrata* cells were isolated as described earlier and used to make a lawn on a YPD agar plate, to the center of which a 6 mm paper disc saturated with 10 μl of 30% H_2_O_2_ (Sigma) was added. A zone of inhibition (diameter of circle with no growth around the disc) was determined by measurement at 24 h. The zone of inhibition reflects the initial resistance of the plated yeast population prior to further growth.

### Phenotypic switching and adhesion assays

Approximately 500 young or old *C*. *glabrata* cells of the parent phenotype were plated on 100 YPD agar plates with 1 mM CuSO_4_, grown for 2 d at 37°C, then stored at 4°C to enhance the switched colony morphology. The ratio of switched to parent colonies was determined over generational age and plotted as the phenotypic switching rate. The switched colonies were streaked for single colonies onto YPD plates three times to determine stability before RLS was determined as described above. Adhesion assays were performed using HeLa cells as described previously [6].

### Antifungal resistance testing

Young or old (5–7 generations old) *C*. *glabrata* cells were isolated as described earlier. Both populations were biotin labeled to allow for isolation after they were allowed to grow in 32 μg/mL fluconazole (Sagent Pharmaceuticals) in YPD broth for 15 h, where old cells could age to 13–15 generations and young cells produced during these 15 hours could be separated from their older mothers. Cells were then washed and the original population of old cells (now 13–15 generations) and the new, young fraction from the originally labeled young population were used in a killing assay as previously described (45). Briefly, cells were diluted to a final concentration of 5 x 10^3^ cells/mL in RPMI 1640 medium buffered with MOPS containing 0–512 μg/mL serial dilutions of fluconazole. 200 μL of each culture was plated in 6 replicates in a 96 well plate and was incubated at 37°C for 4 h. After 4 h, appropriate dilutions were made and plated on YPD plates. Percent inhibition was calculated by comparing CFUs normalized to the CFU from 0 μg/mL fluconazole.

### Chitin and glucan analysis

Chitin composition was analyzed as described previously [87, 88]. For glucan analysis, SDS-treated young or old *C*. *glabrata* cells were separated into alkali-soluble and insoluble fractions, and each fraction was treated with recombinant glucanases and the digested reducing sugars were measured by p-aminohydroxybenzoic acid hydrazine method [89].

### Lipid analysis

Lipids were extracted from young or old *C*. *glabrata* cells using a method adapted from [90]. Briefly, 10^8^ cells for each replicate were pelleted in a glass tube in which mandala extraction buffer was added [91] and extraction was performed. Further extraction was performed per Bligh and Dyer [92] followed by base hydrolysis. The lipid-containing lower organic layer was evaporated to analyze lipids by liquid or gas chromatography-mass spectrometry. For GC-MS, the dried samples were derivatized using *N*,*O*-bis (trimethylsilyl) trifluoroacetamide /trimethylchlorosilane (Sigma-Aldrich) and analyzed using a 30 mt (0.25 μm) VF-5ms column on Agilent 7890 GC-MS (Agilent Technologies, Santa Clara, CA, USA). The retention time of the ergosterol standard (Matreya LLC, PA) was used as a reference. Cholesterol was added as an internal standard for these analyses prior to lipid extraction. LC-MS was performed by the Stony Brook Proteomics core on the samples to quantitate sito and glycosylated ergosterol. A standard curve was generated with sito (R^2^ = 0.991) and used to quantify glycosylated ergosterol.

### Computational modeling of population dynamics

A system of ordinary differential equations was used to model the population dynamics of a *C*. *glabrata* population with distinct age classes over time. For simplicity, the full range of ages was grouped into several classes, namely 0–2, 3–5, 6–8, 9–11 and 12–16 replications (whose population sizes are represented by N_0_, N_1_, N_2_, N_3_ and N_4_, respectively). The free parameters of the model were the mortality rates of each age class, while the replication rates were fixed. Mortality rate profiles were tested for optimality by evaluating the solution of the differential equations analytically at 4 d and compared to the experimental age distribution. This procedure was repeated for many trial sets using a local optimizer, to obtain the mortality profile that gave the global best fit to the experimental data. Additional methods and results are described in Supplemental Information.

### Infection models

*Galleria mellonella* infection was carried out as described previously [61] using a Hamilton syringe to deliver 10^6^ *C*. *glabrata* cells per waxworm. CFU analysis was done by cleaning the waxworm surface with 70% ethanol, puncturing it with a 1 ml syringe, and collecting the haemolymph from 25 worms per time point. Appropriate dilutions were made, plated on YPD agar plates, and incubated at 37°C for 24 h to count CFU.

Murine infections were carried out by intravenously injecting 6–8 week old female Swiss/Webster mice (Taconic) with 7.5 x 10^8^ young or old *C*. *glabrata* cells isolated as described above. One group (n = 15) was injected with the antibody RB6-8C5 [93] every 48 h to deplete neutrophils. Depletion of neutrophils was verified by peripheral cell counts. Mice from both groups were sacrificed (n = 3) at 2 d and 4 d. The fungal burden was collected by homogenizing the kidneys to determine CFU on YPD plates, and budscar count by calcofluor staining (Sigma) as described previously [94].

### *Galleria mellonella* haemocyte collection and phagocytosis assay

Haemocyte collection was performed as previously described [95]. Briefly, haemolymph was collected and diluted 1:10 into Insect Physiological Saline (IPS) [150 mM sodium chloride, 5 mM potassium chloride, 10 mM Tris HCl, 10 mM EDTA and 30 mM sodium citrate] with 10 mM N-ethylmaleimide (anticoagulant). Cells were then centrifuged at 1200 rpm for 10 min, washed with IPS, and counted on a hemocytometer.

For the phagocytosis assay, cells were diluted in RPMI 1640 with 10% Fetal Bovine Serum (FBS). 10^5^ haemocytes were plated in a 96-well plate and incubated at 37°C with 10% CO_2_ overnight to allow for adherence. The following day, non-adherent cells were washed off, and young or old *C*. *glabrata* cells were added to the wells in a 1:50 ratio. The well plate was spun at 1000 rpm for 1 min to ensure contact between haemocytes and yeast, incubated for 1 h to allow for phagocytosis, then washed to remove non-internalized yeast. Yeasts were fixed with cold ethanol for 30 min, and stained with Giemsa for 30 min followed by 3 washes of IPS. Yeasts were analyzed by averaging the number internalized in haemocytes (n = 100).

### RNA sequencing and GO analysis

Three biological replicates of 10^8^ young or old *C*. *glabrata* cells (strain BG2) were isolated, washed and resuspended in 0.5 mm zirconia beads and RLT buffer (Qiagen), then disrupted mechanically using a mini bead beater (Biospec) for 2 min for a total of 4 cycles with 1 min intervals on ice. Post lysis, total RNA was isolated using the RNeasy mini kit (Qiagen) per manufacturer’s instructions. The Genome Technology Access Centre of Washington University in St. Louis (GTAC-WUSTL) performed RNA hybridization, data acquisition and analysis. Briefly, RNA was polyA selected and sequenced on an Illumina HiSeq 2000. Raw sequence reads were converted to basecalls, demultiplexed, and aligned to a reference sequence using Tophat v2.0.9 and Bowtie2 v2.1.0 and gene abundances were derived by HTSeq. Differential expression was estimated by pair-wise negative binomial tests using EdgeR and DEXSeq. Any genes with more than a 2-fold change, a *p* < 0.05 by a hypergeometric test, and an FDR *q <* 0.05 were used to determine significance. Gene ontology (GO) enrichment analysis was done by entering significant genes into the Gene Ontology Term Finder software at http://candidagenome.org/cgi-bin/GO/goTermFinder. The data was deposited at NCBI and can be accessed on GEO (accession #GSE85985).

### Statistics

Standard statistical analysis and non-parametric tests, such as Student’s t-test, Log-rank, one way ANOVA, and Wilcoxon rank sum tests were performed using Prism version 6 (Graphpad) or Microsoft Excel 2011 for Macintosh. Differences are marked with asterisks and considered significant if *p* < 0.05 (*), *p <* 0.01 (**), *p <* 0.001 (***), *p <* 0.0001 (****).

## Supporting information

S1 Fig Enhanced resilience of older *C*. *glabrata* cells was observed in strain BG2. (A) Younger cells were phagocytosed by *Galleria mellonella* haemocytes (B) and human neutrophils at a higher frequency compared to old cells. **P < 0.01, ****P < 0.0001.
(TIF)

S2 Fig Enhanced resilience of older *C*. *glabrata* cells was observed in strains 89 and 117. Increased virulence in *Galleria* was observed with older cells of strains (A) 89 and (B) 117. (C) Younger cells were phagocytosed by *Galleria mellonella* haemocytes at a higher frequency compared to old cells of strain 89. (D) Increased resistance to neutrophil-mediated killing was observed in older cells of strains 89 and 117. (E) H_2_O_2_ disc diffusion assays showed smaller zone of inhibition in older cells of strain 89. (F) The DB variant exhibited a higher fungal burden than the parent (S) in strain 89. (G) Phenotypic switching in strain 89 from S to DB colony morphology increased consistently with age to 18 fold. (H) The RLS of DB was shortened over 50% relative to S and reconstituted in the revertant colony in strain 89. *P < 0.05, **P < 0.01, ***P < 0.001.
(TIF)

S3 Fig Older *C*. *glabrata* cells accumulate *in vivo*. (A) Budscar staining of samples from urine of 5 patients showed a skewed distribution towards an increased frequency of older cells compared to their *in vitro* cultures. (B) Strain 89 cells with a proportionally high number of budscars (mean line) were found at days 2 and 4 in kidneys of WT compared to neutropenic mice, and also compared to day 0.
(TIF)

S4 Fig Dynamics of the populations of each replicative age class over time. Solutions of ordinary differential system with optimum-fit mortality profile for (A) WT mouse host, (B) neutropenic mouse host, and (C) *in vitro* control population.
(TIF)

S5 Fig Dynamics of the populations of each replicative age class over time. Mean of 1000 stochastic simulations of corresponding system of reactions with optimum-fit mortality profile for (A) WT mouse host, (B) neutropenic mouse host, and (C) *in vitro* control population.
(TIF)

S6 Fig Global optimization of mortality rates. Mortality profiles found using a local optimizer are shown for the entire search space of trial profiles. Each locally-optimized mortality profile is marked as being within +/- 20% of the global optimum or not, and its cost function (distance of model from data age distribution, as computed via Eq. (S2) is shown). (A) WT mouse host, (B) neutropenic mouse host, (C) *in vitro* control cell population.
(TIF)

S1 Video Increased budscars by calcofluor staining and cell size observed *in vitro* (strain BG2). (MP4)

S1 Table Kidney burden data for mice infected with strains BG2 or 89. (DOCX)

S1 File Computational modeling of population dynamics. Supplemental methods and results for modeling are described separately.
(PDF)
