# Supplementary figures and images for: Generational distribution of a Candida glabrata population: Resilient old cells prevail, while younger cells dominate in the vulnerable host
Source: PLoS Pathog. 2017 May 10;13(5):e1006355. doi: 10.1371/journal.ppat.1006355 (PMC5440053; doi:10.1371/journal.ppat.1006355)

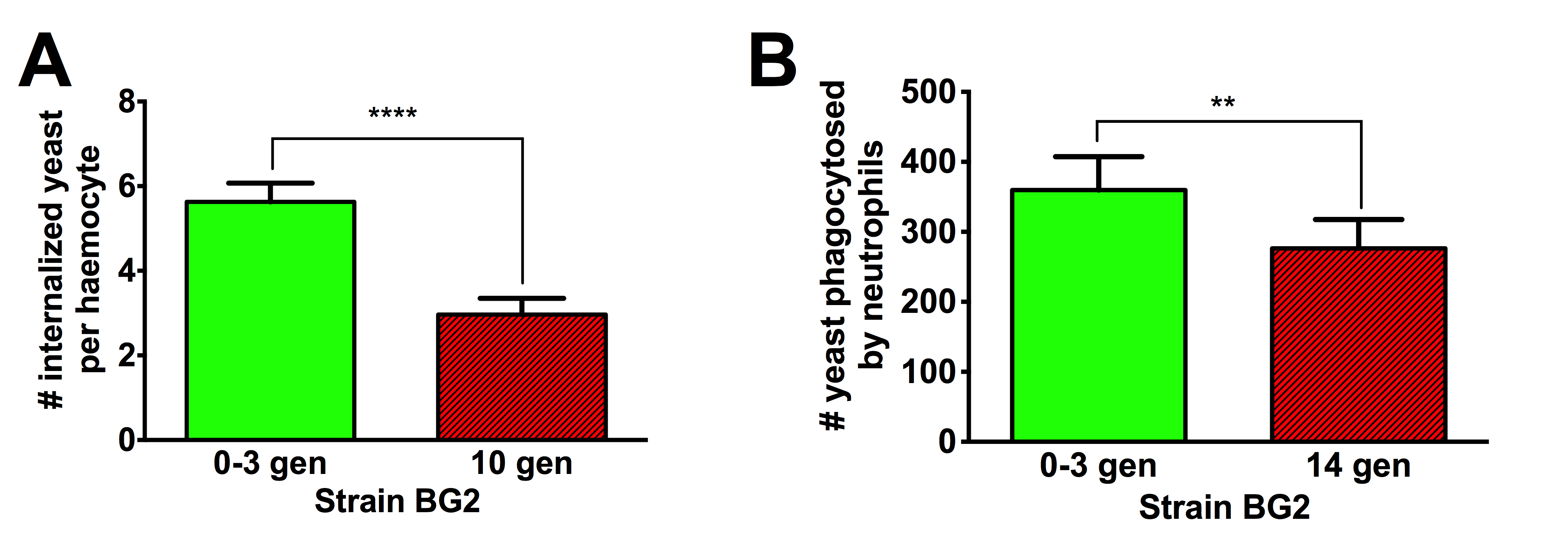

Supplement: S1 Fig — (A) Younger cells were phagocytosed by Galleria mellonella haemocytes (B) and human neutrophils at a higher frequency compared to old cells. **P < 0.01, ****P < 0.0001. (TIF) [file ppat.1006355.s001.tif]

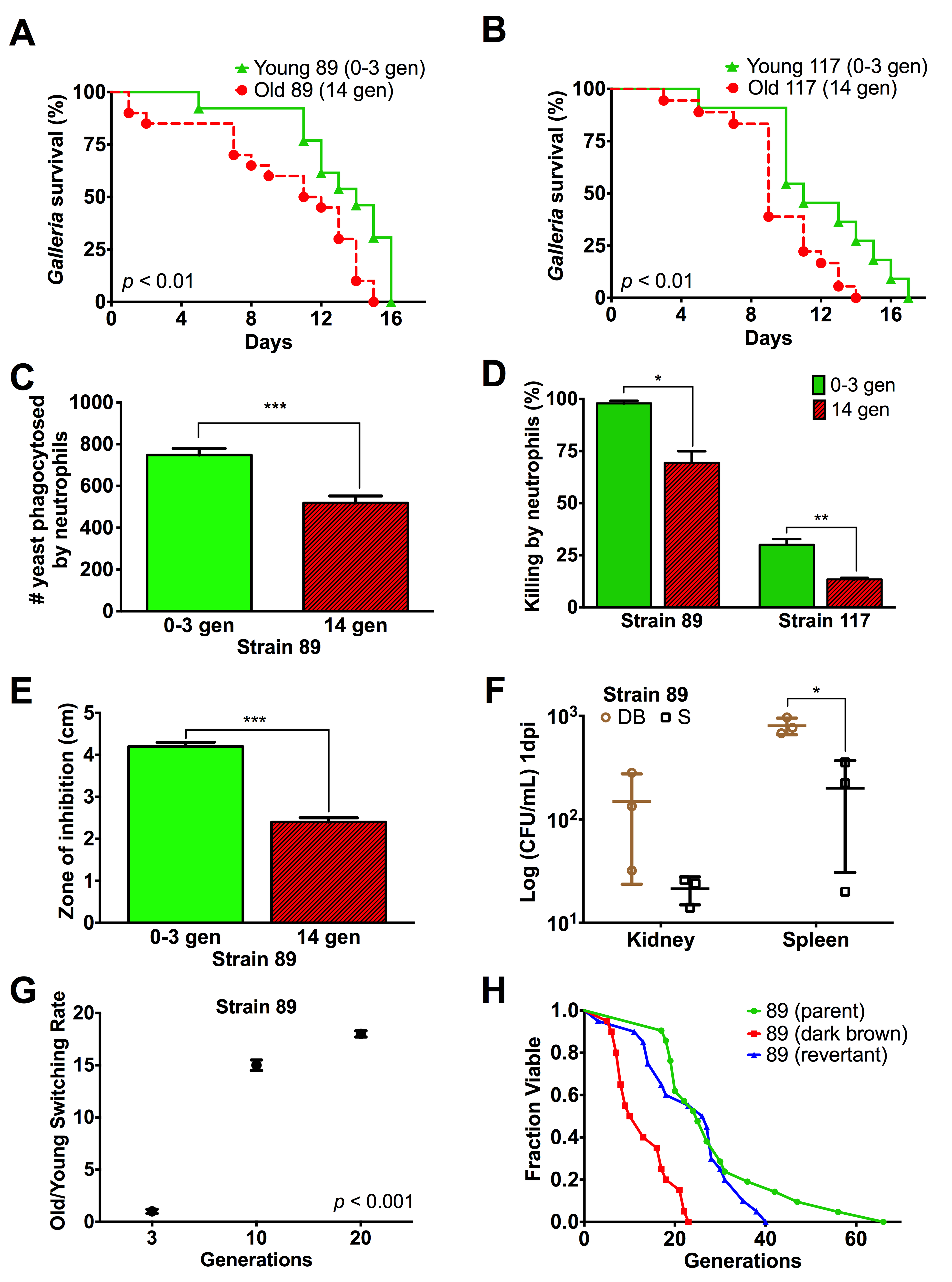

Supplement: S2 Fig — Increased virulence in Galleria was observed with older cells of strains (A) 89 and (B) 117. (C) Younger cells were phagocytosed by Galleria mellonella haemocytes at a higher frequency compared to old cells of strain 89. (D) Increased resistance to neutrophil-mediated killing was observed in older cells of strains 89 and 117. (E) H2O2 disc diffusion assays showed smaller zone of inhibition in older cells of strain 89. (F) The DB variant exhibited a higher fungal burden than the parent (S) in strain 89. (G) Phenotypic switching in strain 89 from S to DB colony morphology increased consistently with age to 18 fold. (H) The RLS of DB was shortened over 50% relative to S and reconstituted in the revertant colony in strain 89. *P < 0.05, **P < 0.01, ***P < 0.001. (TIF) [file ppat.1006355.s002.tif]

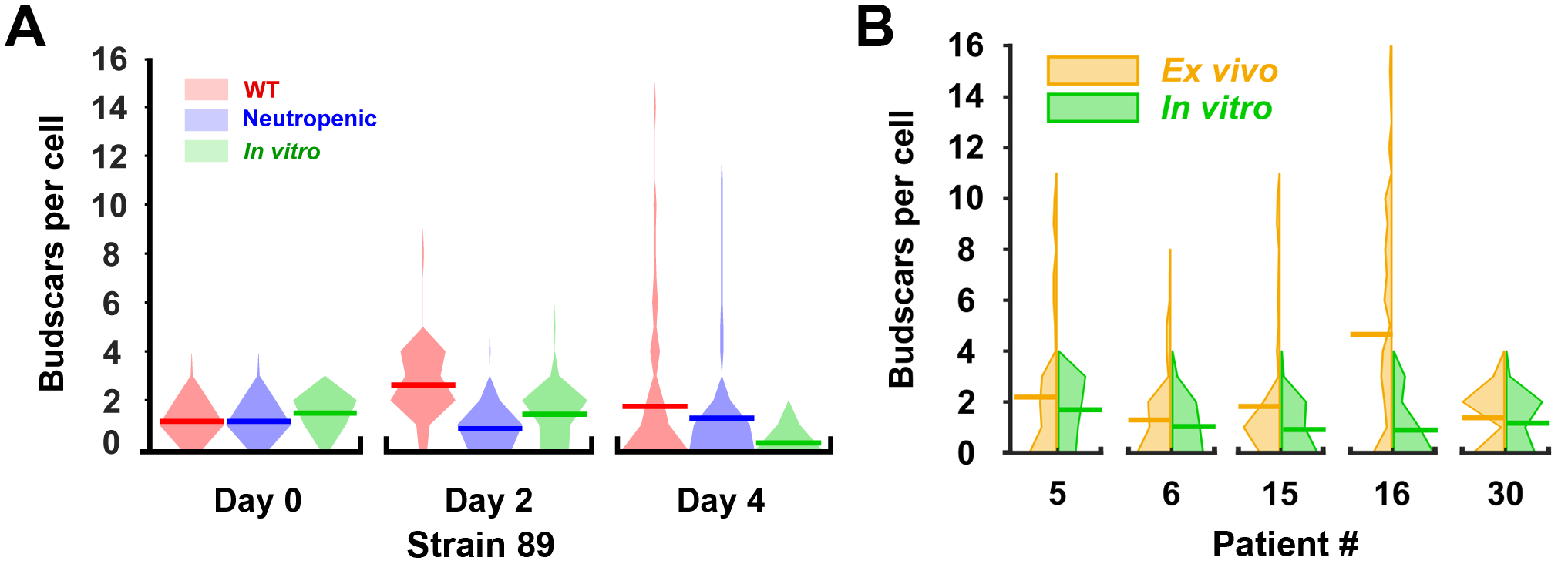

Supplement: S3 Fig — (A) Budscar staining of samples from urine of 5 patients showed a skewed distribution towards an increased frequency of older cells compared to their in vitro cultures. (B) Strain 89 cells with a proportionally high number of budscars (mean line) were found at days 2 and 4 in kidneys of WT compared to neutropenic mice, and also compared to day 0. (TIF) [file ppat.1006355.s003.tif]

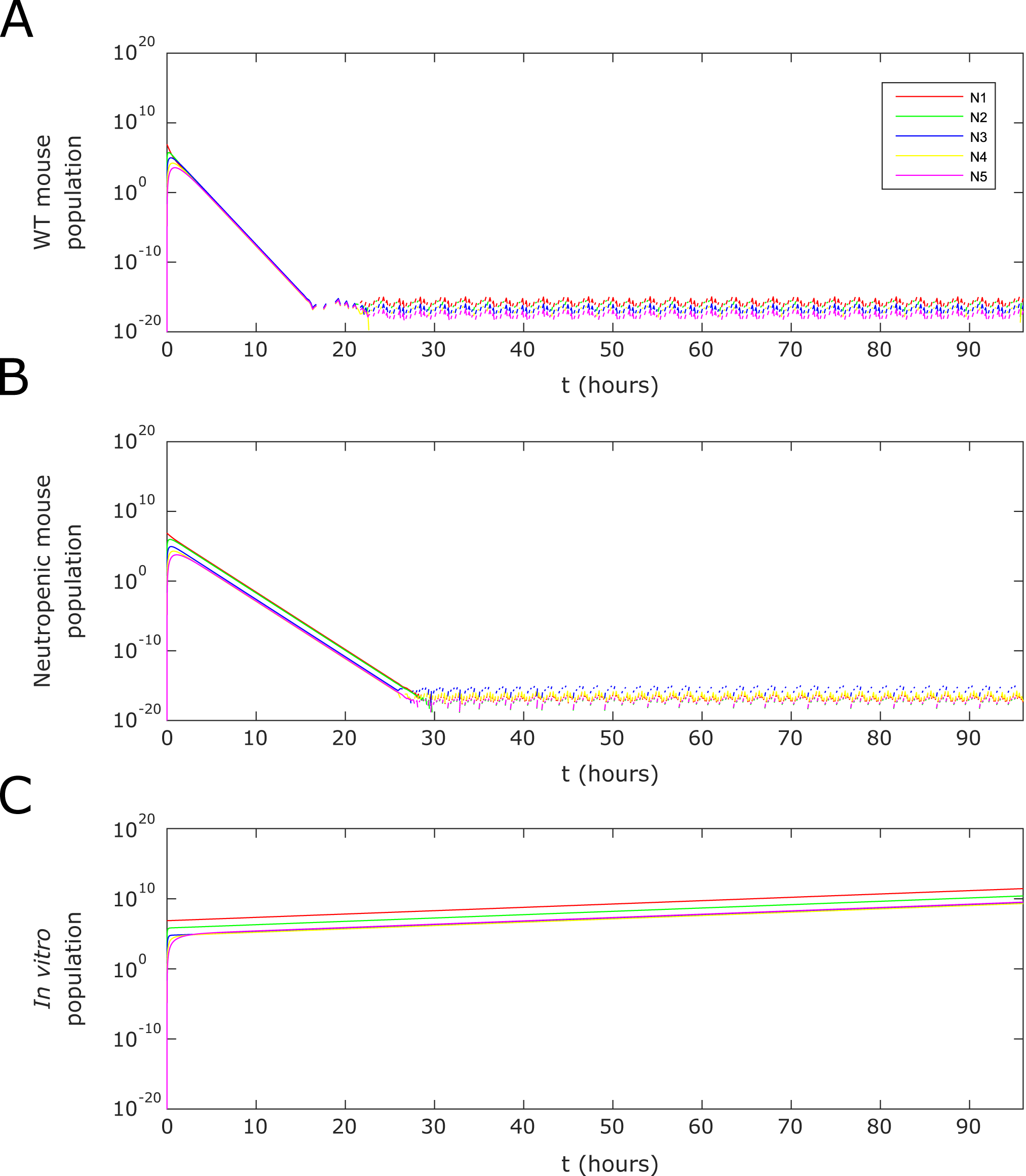

Supplement: S4 Fig — Solutions of ordinary differential system with optimum-fit mortality profile for (A) WT mouse host, (B) neutropenic mouse host, and (C) in vitro control population. (TIF) [file ppat.1006355.s004.tif]

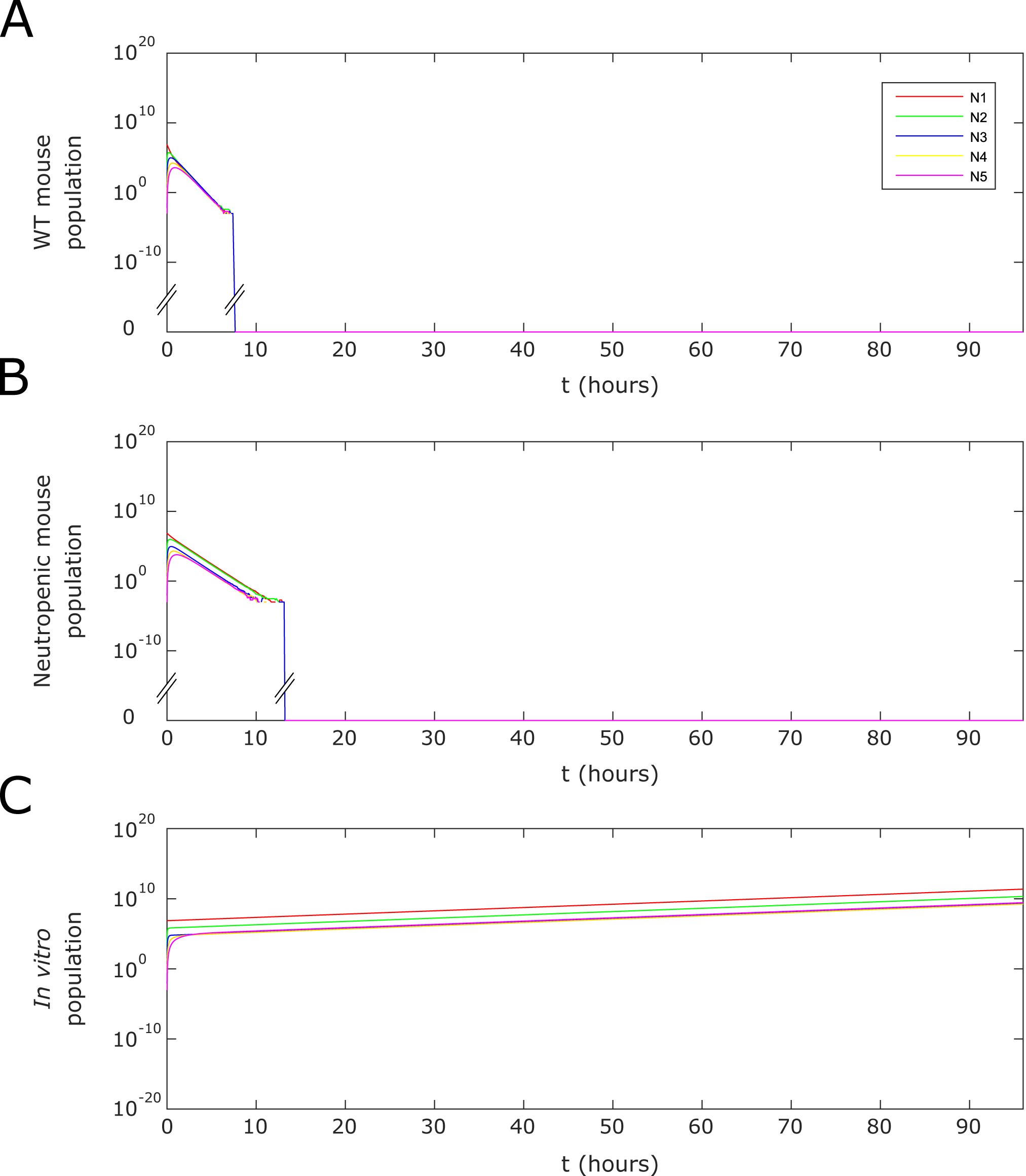

Supplement: S5 Fig — Mean of 1000 stochastic simulations of corresponding system of reactions with optimum-fit mortality profile for (A) WT mouse host, (B) neutropenic mouse host, and (C) in vitro control population. (TIF) [file ppat.1006355.s005.tif]

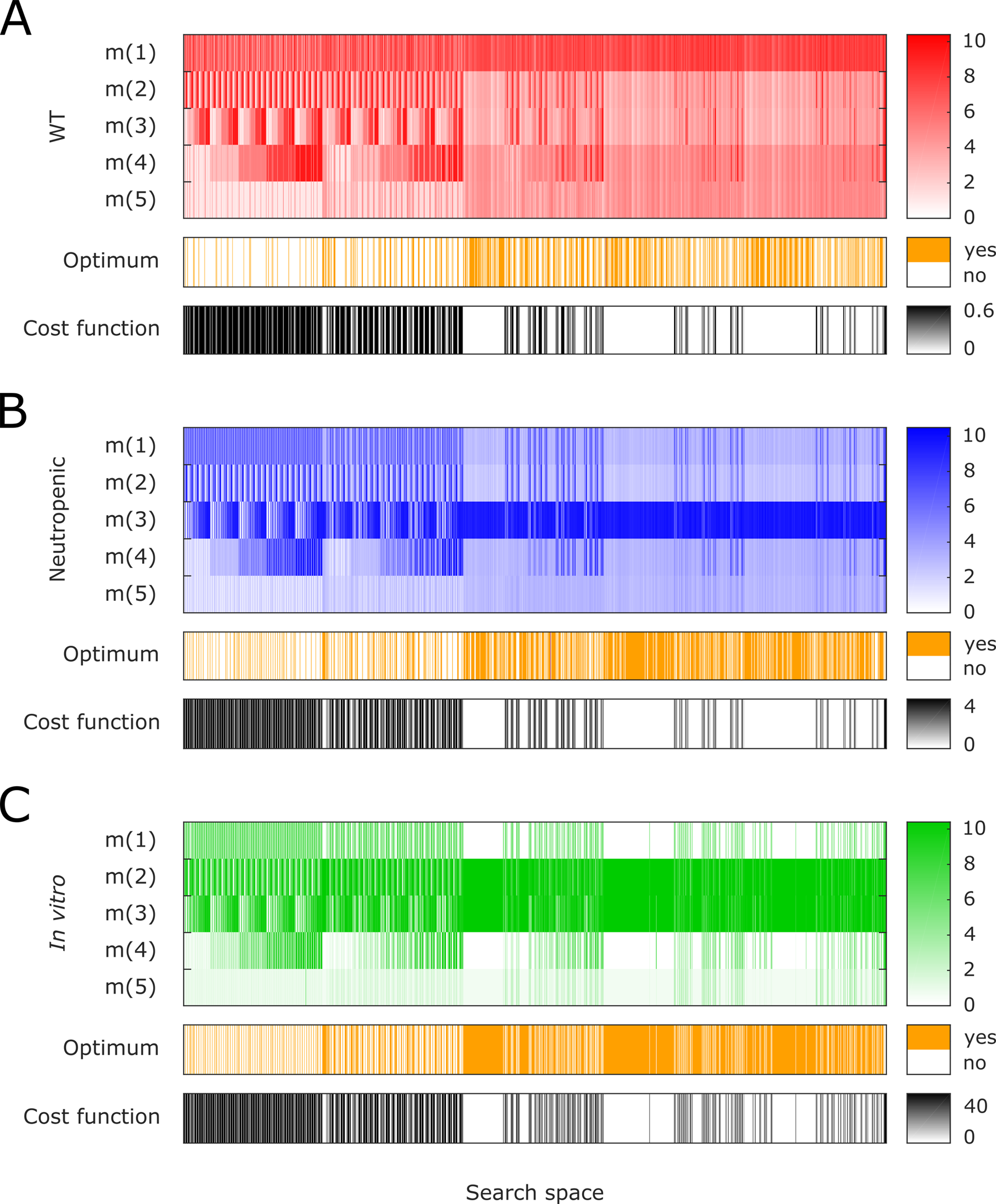

Supplement: S6 Fig — Mortality profiles found using a local optimizer are shown for the entire search space of trial profiles. Each locally-optimized mortality profile is marked as being within +/- 20% of the global optimum or not, and its cost function (distance of model from data age distribution, as computed via Eq. (S2) is shown). (A) WT mouse host, (B) neutropenic mouse host, (C) in vitro control cell population. (TIF) [file ppat.1006355.s006.tif]
