# Supplementary material for: Generational distribution of a Candida glabrata population: Resilient old cells prevail, while younger cells dominate in the vulnerable host
Source: PLoS Pathog. 2017 May 10;13(5):e1006355. doi: 10.1371/journal.ppat.1006355 (PMC5440053; doi:10.1371/journal.ppat.1006355)
Supplement: S1 Table — (DOCX) [file ppat.1006355.s008.docx]

|  | **Day 0** | | | **Day 2** | | | **Day 4** | | |
| --- | --- | --- | --- | --- | --- | --- | --- | --- | --- |
|  | *In vitro* | Wt  mice | Neutropenic  mice | *In vitro* | Wt  mice | Neutropenic  mice | *In vitro* | Wt  mice | Neutropenic  mice |
|  | 7.4 x log 7 | 7.4 x log 7 | 7.4 x log 7 | 2.4 x log 10 | 3.1 x log 3 | 2.1 x log 4 | 4.4 x log 13 | 3.0 x log 3 | 1.4 x log 4 |
| Cells analyzed BG2 | 200 | 200 | 200 | 200 | 200 | 200 | 200 | 200 | 200 |
| Cells analyzed #89 | 158 | 119 | 119 | 142 | 101 | 112 | 99 | 101 | 101 |
