# Supplementary material for: Generational distribution of a Candida glabrata population: Resilient old cells prevail, while younger cells dominate in the vulnerable host
Source: PLoS Pathog. 2017 May 10;13(5):e1006355. doi: 10.1371/journal.ppat.1006355 (PMC5440053; doi:10.1371/journal.ppat.1006355)
Supplement: S1 File — Supplemental methods and results for modeling are described separately. (PDF) [file ppat.1006355.s009.pdf]

1 Methods

We constructed a system of $M+1$ ordinary differential equations to model the populations of *C. glabrata* cells in each age class. The variables $N_i, i = 0,\ldots,M$ (hereafter referred to as $\boldsymbol{N}$, where bold font is used to indicate a vector or matrix) corresponded to the population size (number of cells) of each replicative age class. In the full model, with an arbitrary number of age classes, the population started with N_0 cells in state 0 (virgin cells), some of which proceeded to state 1, then state 2, and so on up to state M as the cells replicated (Eqs. S1). Each replication event also creates a new virgin cell, and cells in every age group can also die.

$$\begin{aligned} \frac{dN_0}{dt} &= \sum_{j=1}^{M-1} r_j N_j - m_0 N_0, \\ \frac{dN_j}{dt} &= r_{j-1}N_{j-1} + r_j N_j - m_j N_j, \\ \frac{dN_M}{dt} &= r_{M-1}N_{M-1} - m_M N_M, \end{aligned} \tag{S1}$$

where N_0, $N_j (j = 1, ..., M-1)$ and N_M (altogether, $N_i, i = 0,\ldots,M$) are the cell population sizes of each replicative age class, r_i are the replication rates, and m_i are the mortality rates of each age class. The time units used in the model were hours, and our known parameters were the replication rates. These were calculated based on experimentally-determined (*in vitro*) doubling times of 40 minutes up to a replicative age of 14, and 65 minutes thereafter (shown in Fig.1C). Since the cells we found experimentally never exceeded 20 replications, we only used these two values for the doubling times. Replication rates were then calculated using the formula $\boldsymbol{r} = \frac{\ln(2)}{\text{doubling time in hours}}$ to give per hour rates. Since the equations are all linear with respect to $\boldsymbol{N}$, this model could be solved both analytically and numerically.

The aim of our computational modeling was to find the optimum set of

mortality parameters $\boldsymbol{m} = m_i, i = 0, \ldots, M$, meaning the set that, when used in our differential equation model (Eqs. S1), would best reproduce the distribution of replicative ages from our mouse experiments (Fig.3A). In order to achieve this, we tested many sets of $\boldsymbol{m}$ (referred to as mortality profiles in the main text and henceforth) and compared the resulting age distributions to Fig.3A. This is a process of optimization; guessing an initial trial mortality profile, we ran a local optimizer to find the optimum profile given this initial guessed profile. However, this only finds a local optimum, and in order to find the global optimum it is necessary to run a local optimizer on the full parameter space of mortality profiles, which we chose to vary from zero to 10 times the replication rate of each age class.

For the local optimization, we used the function *fmincon()* in the Matlab programming environment, which is a constrained optimizer that accepts non-linear, multivariate functions. This takes a set of initial conditions and, using an interior point method, outputs the local optimum of a supplied function (known, for the case of minimization, as the cost function) given these initial conditions. In our case, the initial conditions were trial mortality profiles, and the cost function was given by the closeness of the computationally-found replicative age distribution of *C. glabrata* cells to the experimentally-found one. First, we calculated the values of $\boldsymbol{N}$ (the populations of cells in each replicative age class) using the trial mortality profile, (and the known, standard replication rates $\boldsymbol{r}$) using the matrix equation

$$\boldsymbol{N} = \exp(\boldsymbol{\nu} \times \boldsymbol{C} \times t) \times \boldsymbol{N}_{init},$$

where $\boldsymbol{\nu}$ is the stoichiometric matrix and $\boldsymbol{C}$ is the reaction rate matrix given by the structure of the ordinary differential equation problem. As an example, for the case of $M = 4$, we have five age classes and thus five ordinary

differential equations, given by Eqs. S1 to be

$$\frac{dN_0}{dt} = r_1N_1 + r_2N_2 + r_3N_3 - m_0N_0,$$
$$\frac{dN_1}{dt} = r_0N_0 + r_1N_1 - m_1N_1,$$
$$\frac{dN_2}{dt} = r_1N_1 + r_2N_2 - m_2N_2,$$
$$\frac{dN_3}{dt} = r_2N_2 + r_3N_3 - m_3N_3,$$
$$\frac{dN_4}{dt} = r_3N_3 - m_4N_4.$$

Then the stoichiometric and reaction rate matrices $\boldsymbol{\nu}$ and $\boldsymbol{C}$ are given by

$$\boldsymbol{\nu} = \begin{pmatrix} 0 & 1 & 1 & 1 & -1 & 0 & 0 & 0 & 0 \\ 1 & -1 & 0 & 0 & 0 & -1 & 0 & 0 & 0 \\ 0 & 1 & -1 & 0 & 0 & 0 & -1 & 0 & 0 \\ 0 & 0 & 1 & -1 & 0 & 0 & 0 & -1 & 0 \\ 0 & 0 & 0 & 1 & 0 & 0 & 0 & 0 & -1 \end{pmatrix}$$

and

$$\boldsymbol{C} = \begin{pmatrix} r_0 & 0 & 0 & 0 & 0 \\ 0 & r_1 & 0 & 0 & 0 \\ 0 & 0 & r_2 & 0 & 0 \\ 0 & 0 & 0 & r_3 & 0 \\ m_0 & 0 & 0 & 0 & 0 \\ 0 & m_1 & 0 & 0 & 0 \\ 0 & 0 & m_2 & 0 & 0 \\ 0 & 0 & 0 & m_3 & 0 \\ 0 & 0 & 0 & 0 & m_4 \end{pmatrix}.$$

Now, t is the time of evaluation, chosen as four days to agree with our experiments, and $\boldsymbol{N}_{init}$ gives the initial populations of each replicative age group, chosen as 7.5×10^6 cells spread equally among age groups of zero

to two replications, again to correspond to our experiments. Second, we normalized both the $\boldsymbol{N}$ calculated computationally and observed experimentally at day four (Fig.3A) by dividing their values by the total area of their histograms (i.e., the total number of cells), to convert them into probability distributions of age classes (now labelled $\boldsymbol{p}^N$ and $\boldsymbol{p}^E$, respectively). Finally, we defined our cost function to be a comparison of $\boldsymbol{p}^N$ to $\boldsymbol{p}^E$ using Pearson's chi-squared metric, defined as

$$\text{cost} = \sum_{i=0}^{M} \left(\frac{(p_i^N - p_i^E)^2}{p_i^E + \epsilon} \right), \tag{S2}$$

where $\epsilon = 0.001$ is a workaround to eliminate problems due to division by zero from replicative age classes containing zero cells. Note that bins with zero values do not cause problems, since we are only using Eq. (S2) as a metric of closeness to compare each set of $\boldsymbol{m}$ to each other, rather than as a statistical test. Other possible choices of cost function could have been the L^1 or L^2 metrics.

Thus our global optimization procedure went as follows:

1. Set up ordinary differential equation model (Eqs. S1), choose number of replicative age classes $(M+1)$ and insert parameters $\boldsymbol{r}$ and $\boldsymbol{N}_{init}$.

2. Set up an equally-spaced $(M+1)$-dimensional grid of trial mortality profiles $\boldsymbol{m}$, with $(M+1)^s$ total grid points, where s is the number of points at which we evaluate each of the trial m_i (i.e., each dimension of the total $(M+1)$ dimensions).

3. Parametrize model with trial $\boldsymbol{m}$ chosen from one grid point; calculate cost function using Eq. (S2).

4. Evaluate Step 3 for all $(M+1)^s$ total grid points.

5. Choose global optimum from the set of local optima.

There are two obvious possibilities for the global optimum mortality profile: either the one that gives the overall lowest error (i.e., cost function), or the one that is the most frequent local optimum (these could be one and the same). We wanted our global optimum to be robust, rather than to be a single, possibly unrepresentative, profile, so we examined the frequency of each of the local optima within a certain tolerance of each mortality rate m_i. We set the acceptable bounds as an absolute tolerance of 0.1 (to allow for any of the m_i to be zero) plus a relative tolerance, meaning that m_i should be between the bounds

$$(m_i - \text{abs\_tol}) \times (1 - \tfrac{\text{rel\_tol}}{100}) \leq m_i \leq (m_i + \text{abs\_tol}) \times (1 + \tfrac{\text{rel\_tol}}{100}).$$

Thus, we looked over the entire search space for the mortality profile for which the most other profiles fell within the specified tolerances.

2 Results

Having tested various possibilities for binning the replicative age data to form the reduced model, we settled on five bins (that is, five age groups), containing replicative ages 0-2, 3-5, 6-8, 9-11 and 12-16 (since our experimental results contained cells of maximum age 16, we constrained our computations to this range). This was a result of balancing the improvement in predictive power of our model (by choosing as many bins as possible) with our computational resources (more bins means more computations and thus longer runtime). The doubling times of cells in each of these groups was 40, 40, 40, 40 and 65 minutes, respectively, implying replication rates of $\boldsymbol{r} = 1.04, 1.04, 1.04, 1.04$ and 0.64, respectively. We performed our global optimization over the range $\boldsymbol{m} = [0, 10\boldsymbol{r}]$. Due to computational constraints, we chose a grid size of five, implying a search space of 3125 possible mortality profiles.

In Tables 1 to 3, we show how both the m with overall lowest error and the most frequent m vary with relative tolerance. The m with lowest error remained the same; the relative tolerance did not make a qualitative difference to the most frequent m, except for the unreasonably tolerant case of 75%. Naturally, the frequency of both these measures changed with the relative tolerance. Based on these results, we chose to take as our optimum the mortality profile with lowest error (cost function), as this also had a high frequency (and thus, was a robust result) in all cases. In addition, we chose to use a relative tolerance of 20% for the measure of robustness.

The dynamics of the system of differential equations over time with the optimum parameters are shown in Fig. S2. We used a numerical solver to generate these time courses. Notice that after approximately 10 and 20 hours for wild-type and neutropenic mouse hosts, respectively, the precision of the solution becomes limited by the precision of the solver, as the populations have become so low that they effectively become zero (differential equations such as these will never exactly reach zero, instead moving closer and closer to it indefinitely; evaluating the equations analytically over time would show exactly this).

Relative m	Minimum cost function			Most frequent m		
tolerance	Frequency	m values	Error	Frequency	m values	Mean error
10%	24%	[6.9 3.0 2.8 4.1 4.0]	3.0×10^{-18}	36%	[7.4 3.5 3.3 4.6 4.5]	2.1×10^{-16}
20%	38%	[6.9 3.0 2.8 4.1 4.0]	3.0×10^{-18}	48%	[7.5 3.5 3.4 4.6 4.5]	5.4×10^{-16}
35%	51%	[6.9 3.0 2.8 4.1 4.0]	3.0×10^{-18}	57%	[7.5 3.5 3.4 4.6 4.5]	1.4×10^{-15}
50%	58%	[6.9 3.0 2.8 4.1 4.0]	3.0×10^{-18}	60%	[7.5 3.5 3.4 4.6 4.5]	3.3×10^{-16}
75%	64%	[6.9 3.0 2.8 4.1 4.0]	3.0×10^{-18}	87%	[7.3 5.4 7.0 6.9 3.5]	0.61

Table 1. Effects of varying relative tolerance on mortality profile m (WT mouse host). Frequency to within relative tolerance on m, actual m values and error (given by the cost function as defined by Eq. (S2)) of both the m with lowest error, as well as most frequent m. If more than one set of m fulfilled the latter conditions, the mean of their errors is reported.

Relative m tolerance	Minimum cost function Frequency	m values	Error	Most frequent m Frequency	m values	Mean error
10%	42%	[2.8 2.3 9.3 2.9 2.8]	8.6×10^{-20}	46%	[3.1 2.6 9.6 3.2 3.1]	3.4×10^{-16}
20%	52%	[2.8 2.3 9.3 2.9 2.8]	8.6×10^{-20}	59%	[3.0 2.5 9.6 3.2 3.0]	2.4×10^{-16}
35%	65%	[2.8 2.3 9.3 2.9 2.8]	8.6×10^{-20}	65%	[2.8 2.2 9.3 2.9 2.8]	1.2×10^{-16}
50%	66%	[2.8 2.3 9.3 2.9 2.8]	8.6×10^{-20}	66%	[2.6 2.1 9.1 2.7 2.6]	2.8×10^{-16}
75%	67%	[2.8 2.3 9.3 2.9 2.8]	8.6×10^{-20}	82%	[7.8 7.8 7.8 7.8 3.2]	4.0

Table 2. Effects of varying relative tolerance on mortality profile m (neutropenic host).

Relative m tolerance	Minimum cost function Frequency	m values	Error	Most frequent m Frequency	m values	Mean error
10%	63%	[0.0 10.4 10.4 0.0 0.5]	0.74	63%	[0.0 10.4 10.4 0.0 0.5]	0.74
20%	63%	[0.0 10.4 10.4 0.0 0.5]	0.74	63%	[0.0 10.4 10.4 0.0 0.5]	0.74
35%	63%	[0.0 10.4 10.4 0.0 0.5]	0.74	63%	[0.0 10.4 10.4 0.0 0.5]	0.74
50%	63%	[0.0 10.4 10.4 0.0 0.5]	0.74	63%	[0.0 10.4 10.4 0.0 0.5]	0.74
75%	63%	[0.0 10.4 10.4 0.0 0.5]	0.74	63%	[0.0 10.4 10.4 0.0 0.5]	0.74

Table 3. Effects of varying relative tolerance on mortality profile m (*in vitro*).

We can also write this system of differential equations as a set of reactions, which are also specified by the matrices $\boldsymbol{\nu}$ and $\boldsymbol{C}$. In this case we can simulate the time evolution of these reactions as a stochastic process, using a formalism developed for chemical reactions (1, 2). Now, the population changes randomly over time and no two sets of simulation time courses will be identical. The populations of the stochastic system are given by distributions that change over time. Taking the average of a large number of these simulations (1000 tau-leap simulations with error parameter $\varepsilon = 0.05$) gives us the mean dynamics of the populations over time (Fig. S3).The differential equations and mean of the stochastic simulations agree, which is not surprising since the differential equations are all linear. Note that the mean populations in the stochastic simulations reach zero around seven and 12-13 hours for wild-type and neutropenic mouse hosts, respectively. Just prior to reaching zero, the populations in

each replicative age class are around 10^{-3}; as comparison, the differential equations also reach this value at around seven and 12 hours (for wild-type and neutropenic mouse hosts, respectively); however, this is difficult to tell from the figure.

Finally, an overview of the global optimization that led to these results is shown in Fig. S4. This shows pictorially the robustness of the results in Tables 1 to 3. We can see that as the initial trial m move away from zero, the local optimizer finds local optima that are roughly consistent, as well as decreasing the cost function (implying closeness of fit of our model results to the experiments). Thus we can, at a glance, tell that our global optimization does indeed find a robust global optimum, which we have defined as the mortality profile with the lowest cost function.
